# Supplementary figures and images for: M-Sec promotes the production of infectious HIV-1 virus through the exocyst complex in macrophages
Source: PLoS Pathog. 2026 Jun 1;22(6):e1013717. doi: 10.1371/journal.ppat.1013717 (PMC13235924; doi:10.1371/journal.ppat.1013717)

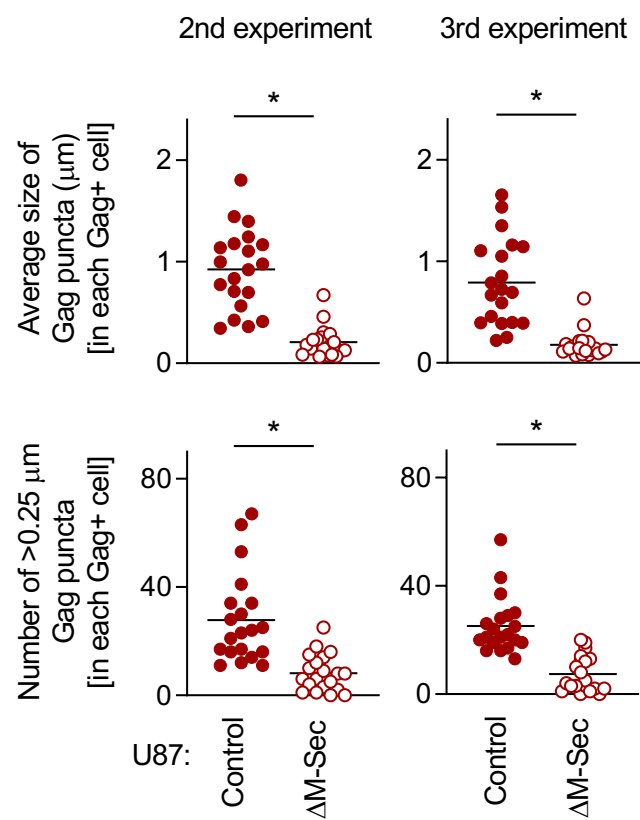

S1 Fig

Supplement: S1 Fig — (PDF) [file ppat.1013717.s001.pdf]

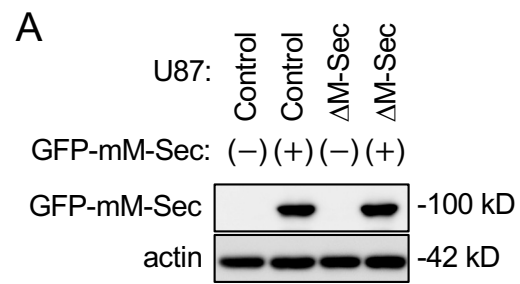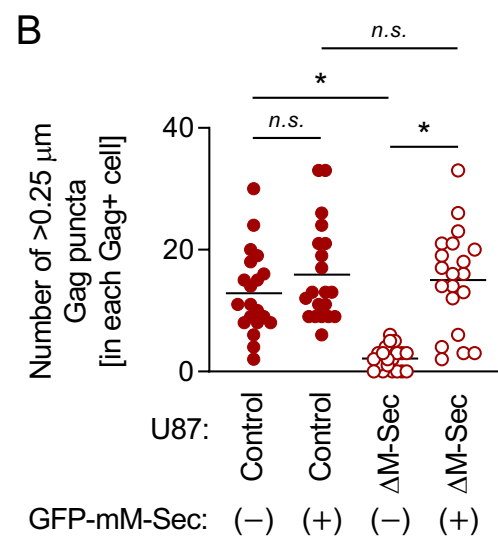

S2 Fig

Supplement: S2 Fig — (A, B) The control or stable M-Sec knockdown (ΔM-Sec) U87 cells were transfected with the empty vector (“−”) or the GFP-fused mouse M-Sec (GFP-mM-Sec) expression plasmid (“+”), and cultured for 3 days. In A, the cells were analyzed for their expression level of GFP-mM-Sec by western blotting. β-actin blot is the loading control. In B, the cells were infected with HIV-1, cultured for 2 days, and analyzed for Gag by immunofluorescence. The number of >0.25 μm Gag puncta in each Gag+ cell is summarized (20 cells for each group). n.s., not significant. *p < 0.05. (PDF) [file ppat.1013717.s002.pdf]

A

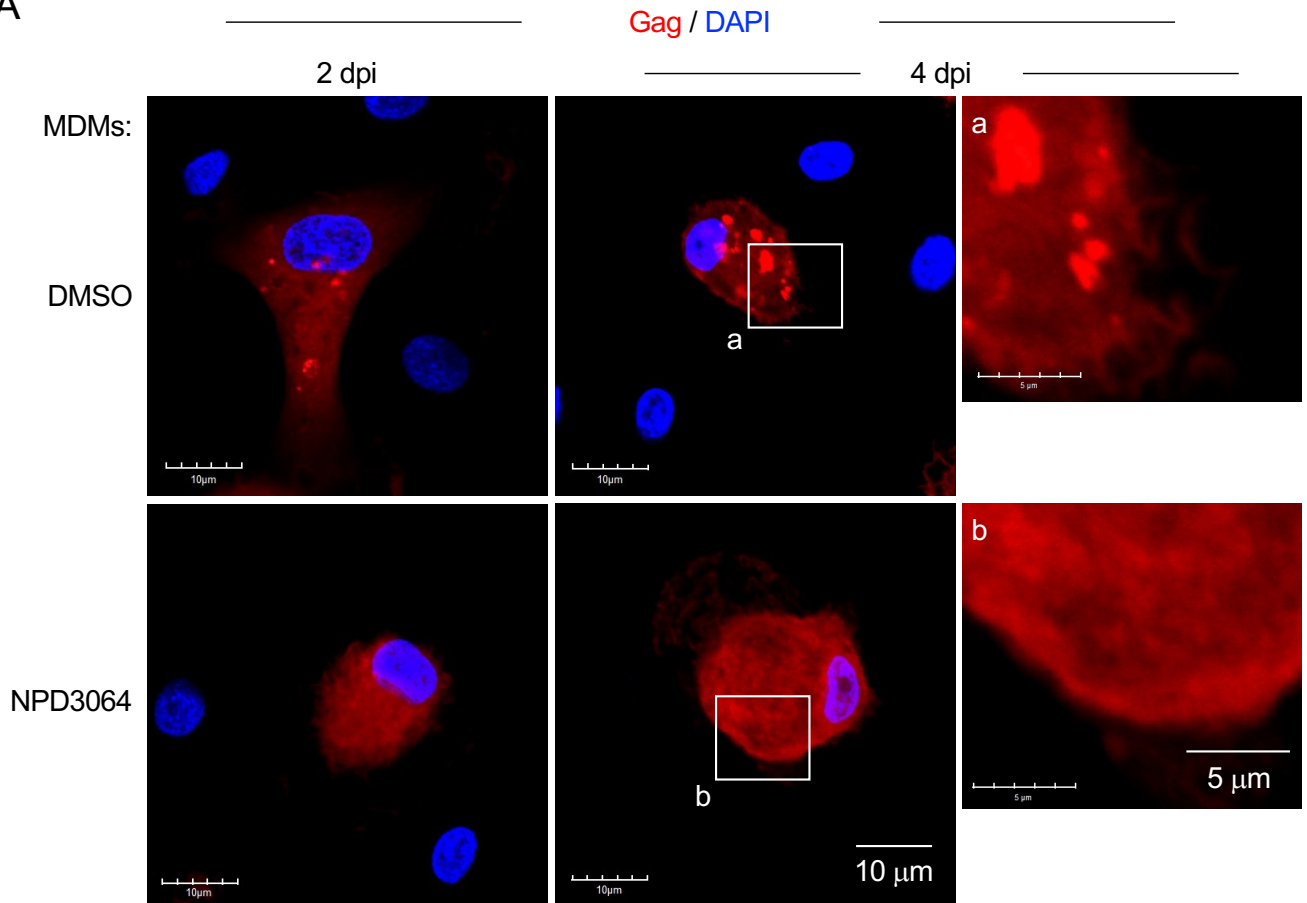

B

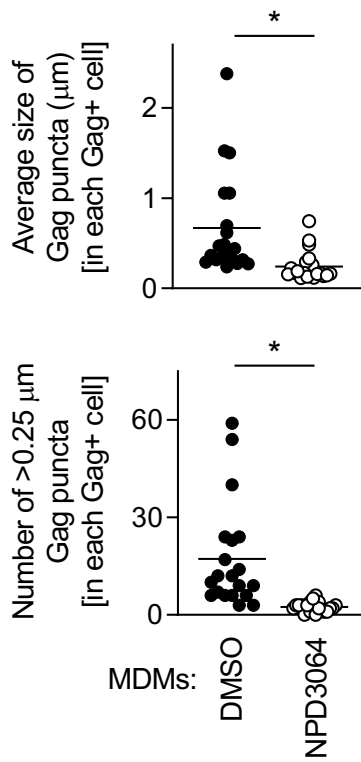

C

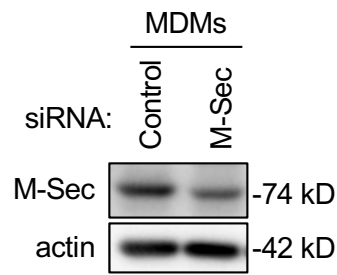

D

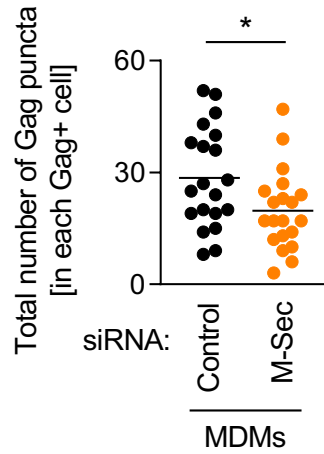

S3 Fig

Supplement: S3 Fig — (A) Peripheral blood monocyte-derived macrophages (MDMs) were pretreated with DMSO or 10 μM NPD3064 for 2 days, and infected with HIV-1. After two or four days of infection (dpi), the cells were analyzed for Gag (red) by immunofluorescence. The nuclei were stained with DAPI (blue). In the right panels, the magnified images of “a” and “b” in the middle panels are shown. Scale bars: 10 μm and 5 μm for the left/middle and right panels, respectively. (B) MDMs were infected for 4 days, and analyzed as in A. In the upper panel, the average size of Gag puncta in each Gag+ cell is summarized (20 cells for each group). In the lower panel, the number of >0.25 μm Gag puncta in each Gag+ cell is summarized (20 cells for each group). The Gag signal larger than approximately 0.03 μm was considered puncta. *p < 0.05. Data shown are a representative of three independent experiments. (C, D) In C, MDMs were transfected with the control siRNA or M-Sec-specific siRNA, cultured for 4 days, and analyzed for their expression level of M-Sec by western blotting. β-actin blot is the loading control. In D, MDMs were transfected with the control siRNA or M-Sec-specific siRNA, and cultured for 2 days. Then, the cells were infected with HIV-1, cultured for 2 days, and analyzed for Gag by immunofluorescence. The total number of Gag puncta (>0.03 μm) in each Gag+ cell is summarized (20 cells for each group). Data shown are a representative of three independent experiments. (PDF) [file ppat.1013717.s003.pdf]

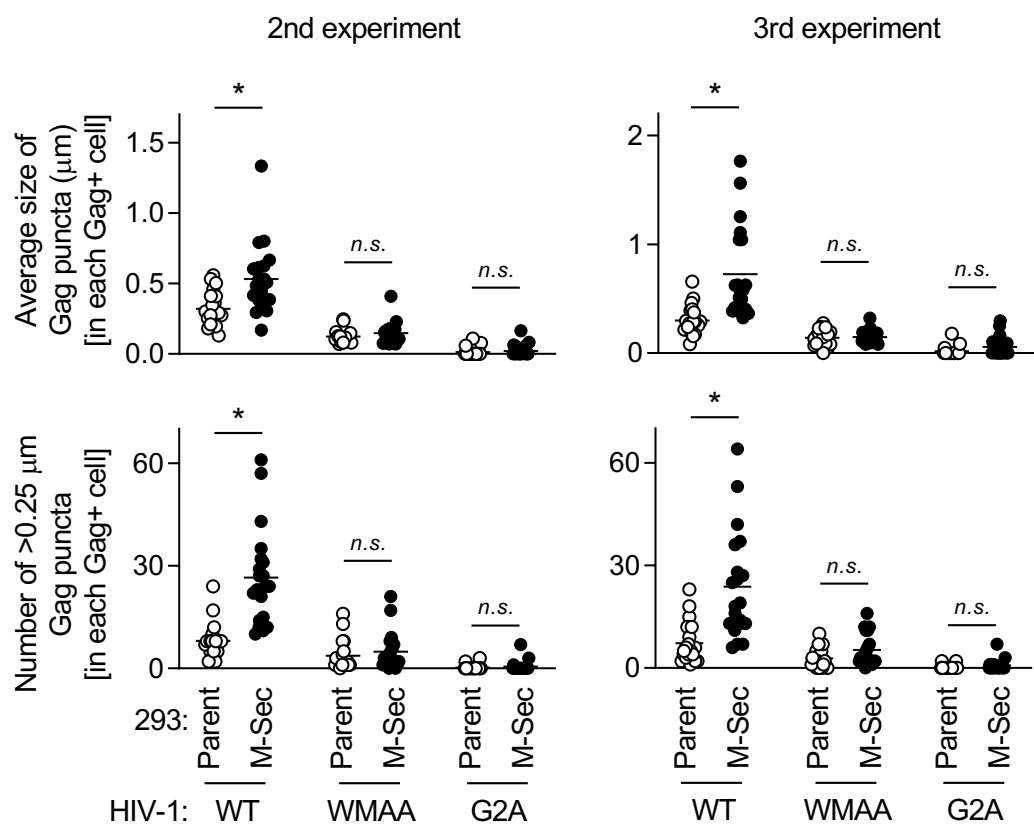

S4 Fig

Supplement: S4 Fig — (PDF) [file ppat.1013717.s004.pdf]

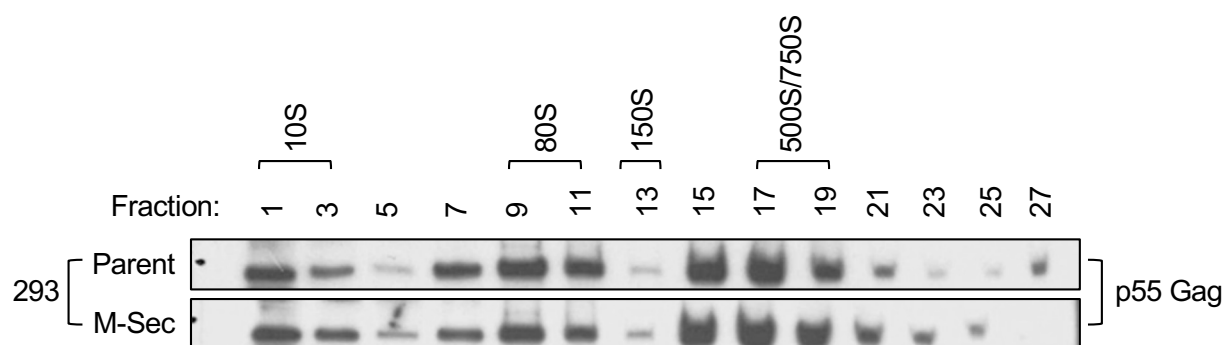

S5 Fig

Supplement: S5 Fig — The parental or stable M-Sec-expressing 293 cells were transfected with the wild-type (WT) HIV-1 molecular clone, and cultured for 2 days. The cell lysates were prepared and ultracentrifuged through sucrose gradients. Fractions were collected from centrifuge tubes (from top to bottom), and analyzed for Gag (p55) by western blotting. Sedimentation coefficients (S values) are also shown. 10S, 80S/150S, and 500S/750S contain nascent-, oligomerized-, and multimerized Gag, respectively [14]. (PDF) [file ppat.1013717.s005.pdf]

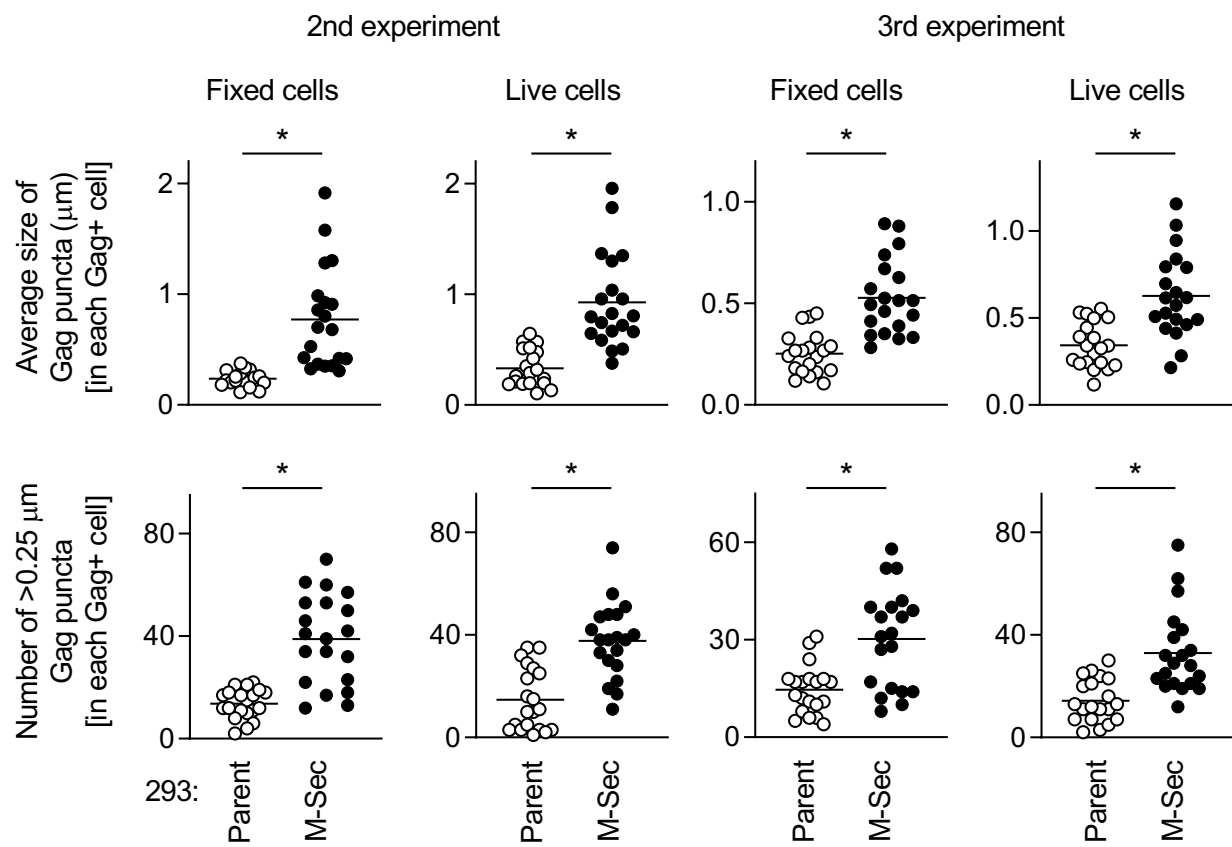

S6 Fig

Supplement: S6 Fig — (PDF) [file ppat.1013717.s006.pdf]

Nef-GFP

Nef-GFP / DAPI

293:

Parent

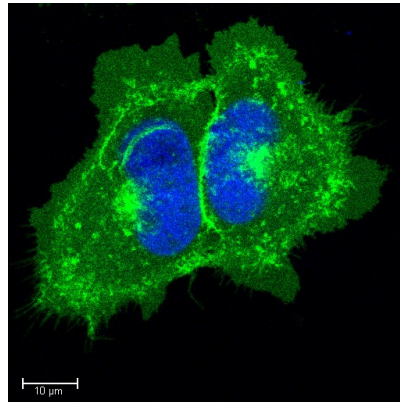

M-Sec

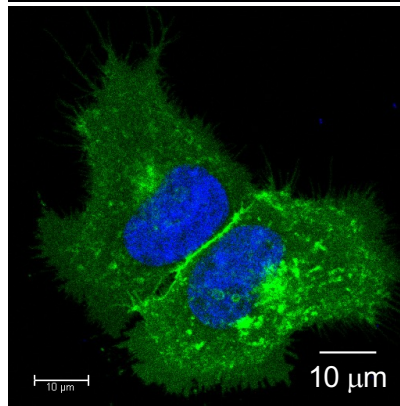

S7 Fig

Supplement: S7 Fig — The parental or stable M-Sec-expressing 293 cells were transfected with the Nef-GFP expression plasmid, cultured for 2 days, and analyzed for Nef-GFP (green) by immunofluorescence. The nuclei were stained with DAPI (blue). Scale bar: 10 μm. (PDF) [file ppat.1013717.s007.pdf]

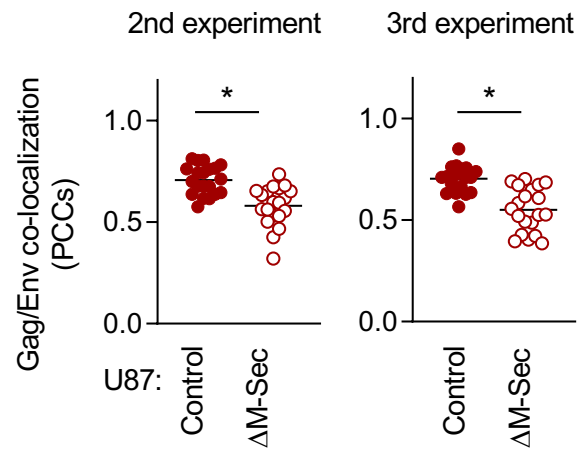

S8 Fig

Supplement: S8 Fig — (PDF) [file ppat.1013717.s008.pdf]

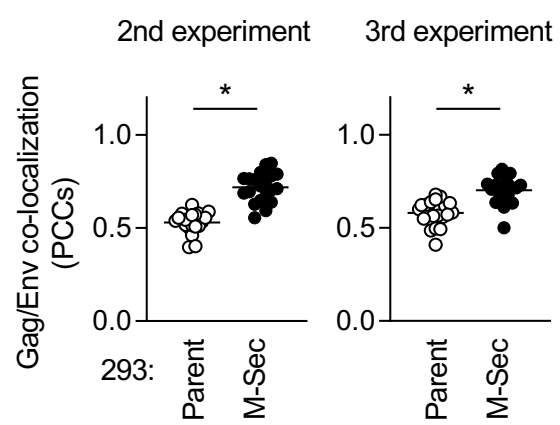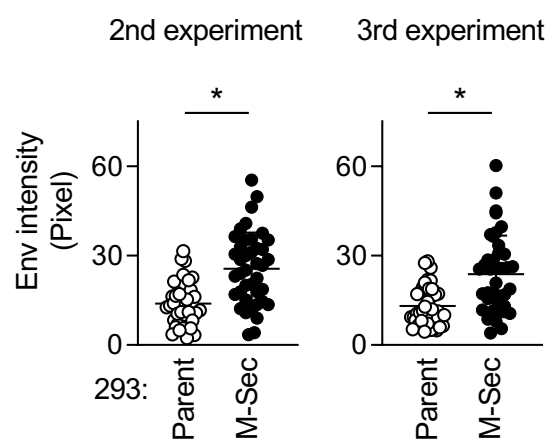

S9 Fig

Supplement: S9 Fig — (PDF) [file ppat.1013717.s009.pdf]

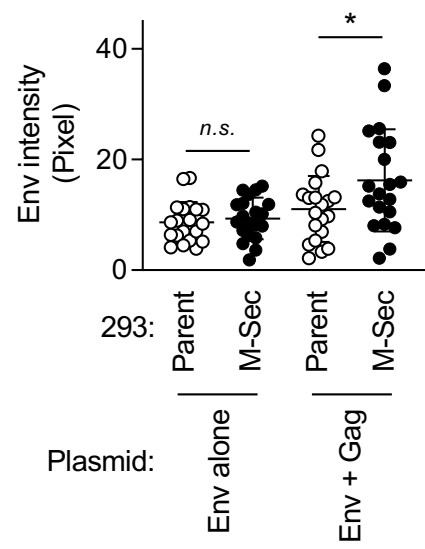

S10 Fig

Supplement: S10 Fig — The parental or stable M-Sec-expressing 293 cells were transfected with the Env expression plasmid (Env alone), or co-transfected with the expression plasmids of Env and Gag (Env + Gag). Then, the cells were cultured for 2 days, and analyzed for Env by immunofluorescence. The density of Env was quantified by randomly selecting Env-positive areas (90 μm2 for each area, two areas for each cell, total 20 areas for each group). n.s., not significant. *p < 0.05. Data shown are a representative of three independent experiments. (PDF) [file ppat.1013717.s010.pdf]

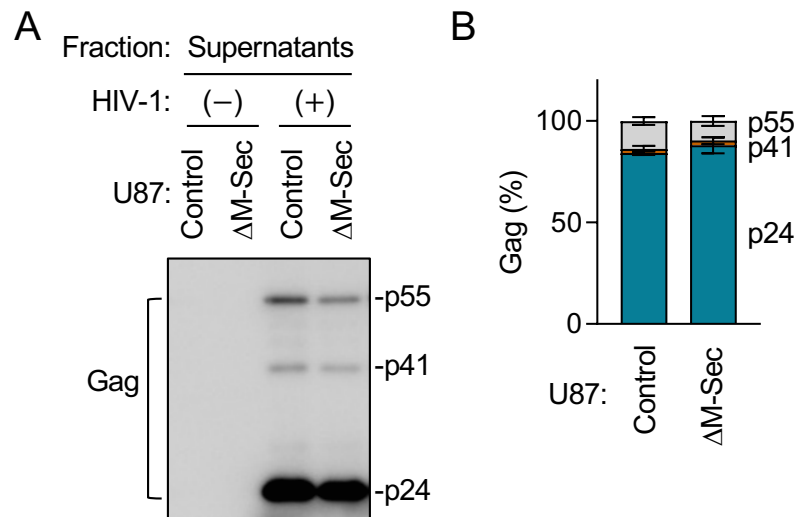

S11 Fig

Supplement: S11 Fig — (A, B) The control or stable M-Sec knockdown (ΔM-Sec) U87 cells were left uninfected or infected with HIV-1, and cultured for 2 days. The virus-like particles in the supernatants were collected by centrifugation, and analyzed for their amount of p55 Gag, p41 Gag, and p24 Gag by western blotting. In B, the percentage of each Gag protein to the total Gag proteins is summarized (n = 3). (PDF) [file ppat.1013717.s011.pdf]

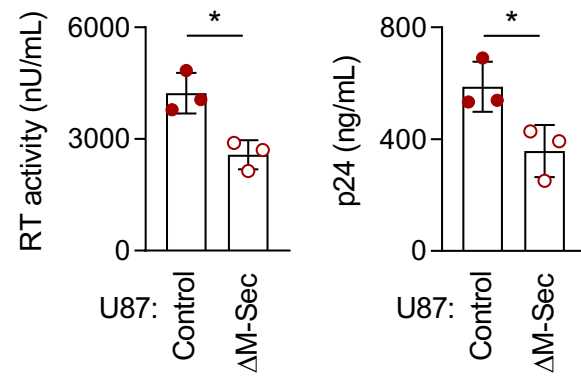

S12 Fig

Supplement: S12 Fig — The control or stable M-Sec knockdown (ΔM-Sec) U87 cells were infected with HIV-1, and cultured for 2 days. The supernatants were collected, and analyzed for the activity of reverse transcriptase (RT) by qPCR (n = 3, left), or p24 Gag by ELISA (n = 3, right). *p < 0.05. (PDF) [file ppat.1013717.s012.pdf]

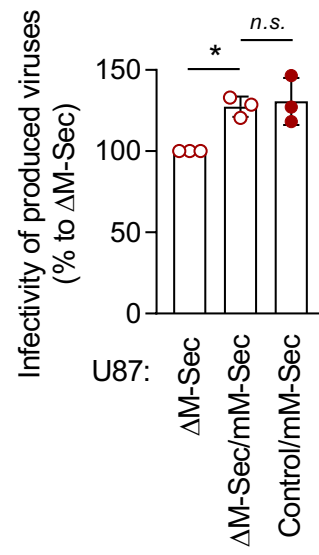

S13 Fig

Supplement: S13 Fig — The stable M-Sec knockdown U87 cells (ΔM-Sec), the ΔM-Sec U87 cells stably expressing mouse M-Sec (ΔM-Sec/mM-Sec), or the control U87 cells stably expressing mM-Sec (Control/mM-Sec) were infected with HIV-1, and cultured for 2 days. The supernatants were collected, and analyzed for viral infectivity using TZM-bl cells as the target cells (the viral input: 2 nU/mL reverse transcriptase activity). The infectivity is represented as a percentage relative to that of the ΔM-Sec U87 cells (n = 3). n.s., not significant. *p < 0.05. (PDF) [file ppat.1013717.s013.pdf]

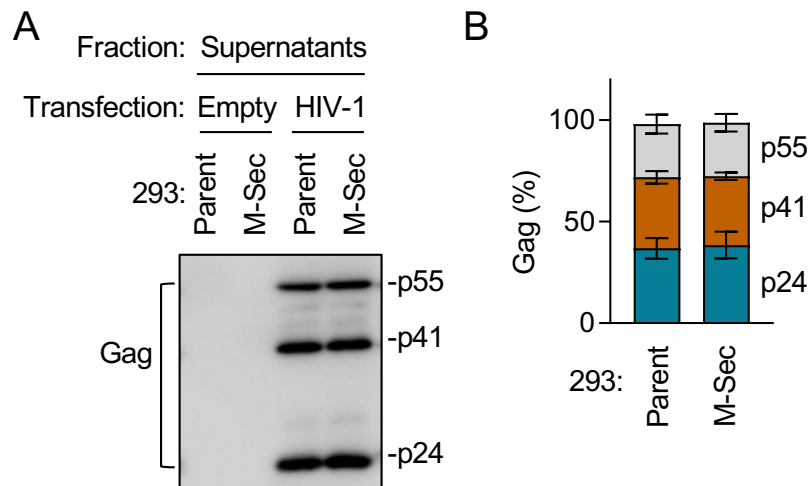

S14 Fig

Supplement: S14 Fig — (A, B) The parental or stable M-Sec-expressing 293 cells were transfected with the empty plasmid or HIV-1 molecular clone, and cultured for 2 days. The virus-like particles in the supernatants were collected by centrifugation, and analyzed for their amount of p55 Gag, p41 Gag, and p24 Gag by western blotting. In B, the percentage of each Gag protein to the total Gag proteins is summarized (n = 3). (PDF) [file ppat.1013717.s014.pdf]

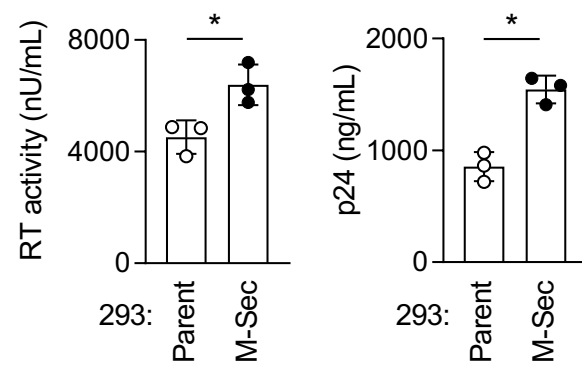

S15 Fig

Supplement: S15 Fig — The parental or stable M-Sec-expressing 293 cells were transfected with the empty plasmid or HIV-1 molecular clone, and cultured for 2 days. The supernatants were collected, and analyzed for the activity of reverse transcriptase (RT) by qPCR (n = 3, left), or p24 Gag by ELISA (n = 3, right). *p < 0.05. (PDF) [file ppat.1013717.s015.pdf]

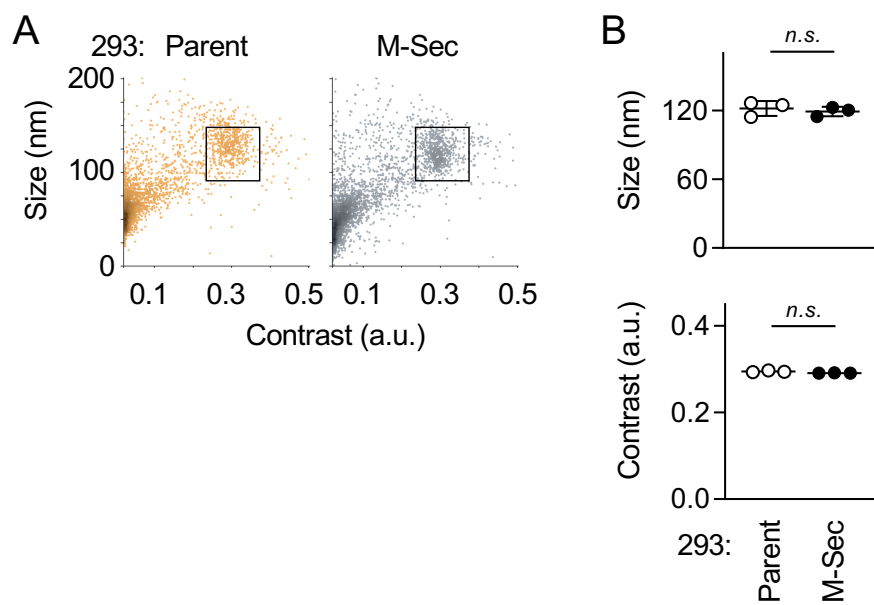

S16 Fig

Supplement: S16 Fig — (A, B) The parental or stable M-Sec-expressing 293 cells were transfected with the empty plasmid or HIV-1 molecular clone, and cultured for 2 days. The virus-like particles in the supernatants were collected by centrifugation, and analyzed for their size and weight (contrast) by mass photometry. In A, a typical example is shown. In B, the results of three independent assays were summarized. a.u., arbitrary unit. n.s., not significant. (PDF) [file ppat.1013717.s016.pdf]

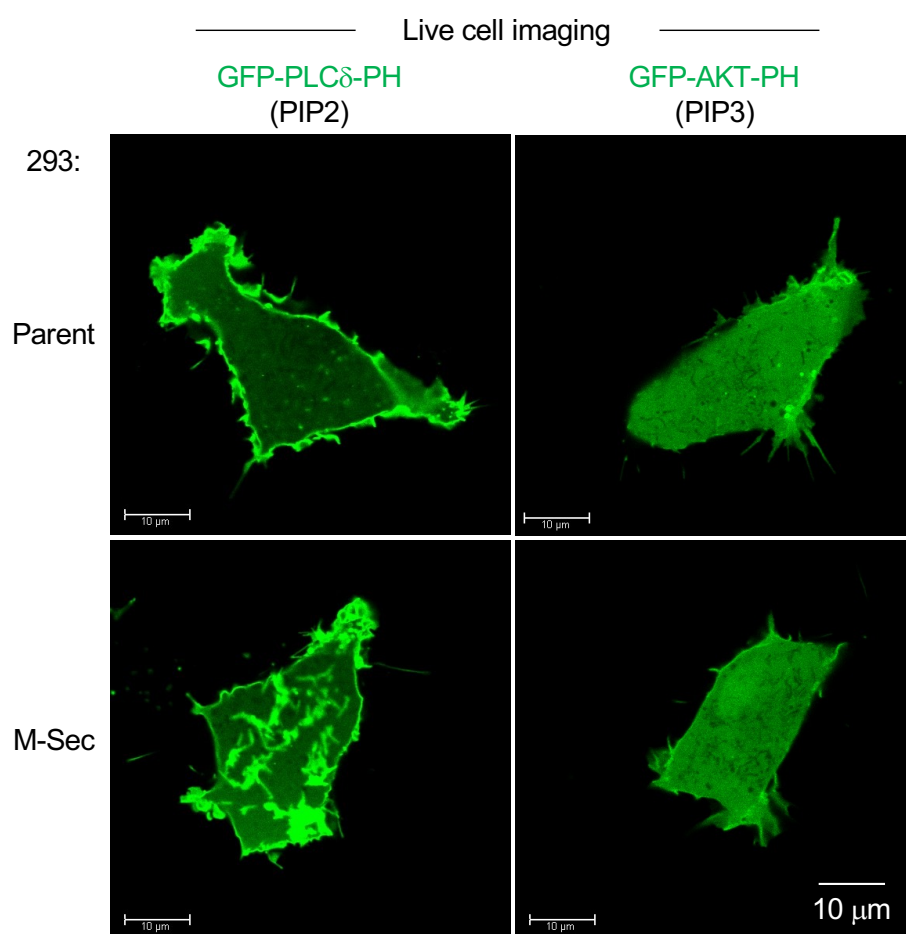

S17 Fig

Supplement: S17 Fig — The parental or stable M-Sec-expressing 293 cells were transfected with the plasmid expressing either GFP-PLCδ-PH (PIP2 probe) or GFP-Akt-PH (PIP3 probe), cultured for 24 hours, and analyzed for the GFP signal by live cell imaging. Scale bar: 10 μm. (PDF) [file ppat.1013717.s017.pdf]

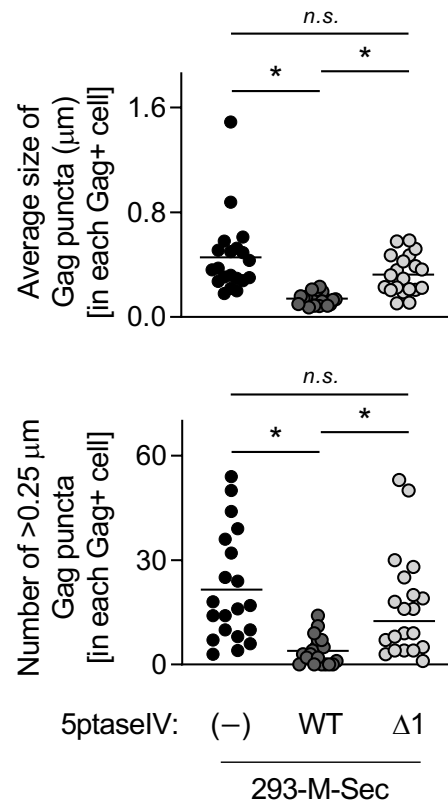

S18 Fig

Supplement: S18 Fig — The M-Sec-expressing 293 cells were co-transfected (1:1) with the Gag-GFP expression plasmid and the empty plasmid (−), or 5ptaseIV plasmid (the wild-type (WT) or defective Δ1 mutant). The cells were cultured for 2 days and analyzed for Gag-GFP by immunofluorescence. In the upper panel, the average size of Gag puncta in each Gag+ cell is summarized (20 cells for each group). In the lower panel, the number of >0.25 μm Gag puncta in each Gag+ cell is summarized (20 cells for each group). The Gag signal larger than approximately 0.03 μm was considered puncta. n.s., not significant. *p < 0.05. (PDF) [file ppat.1013717.s018.pdf]

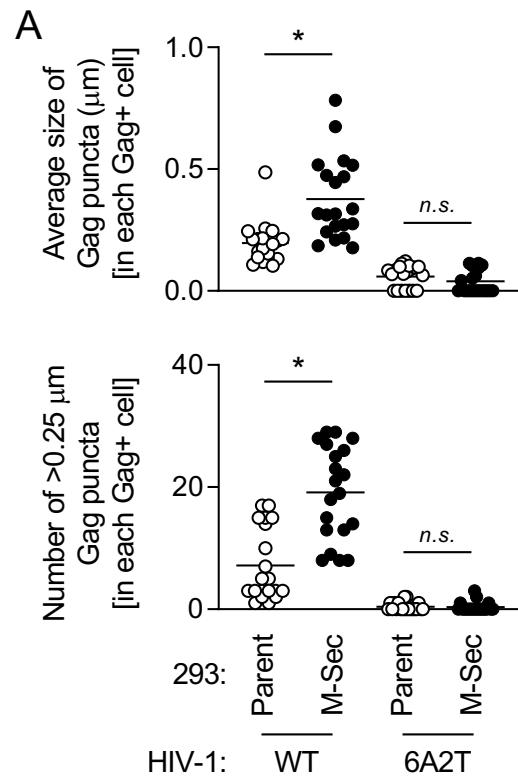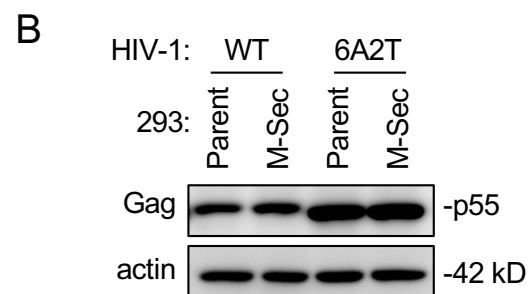

S19 Fig

Supplement: S19 Fig — (A) The parental or stable M-Sec-expressing 293 cells were transfected with the wild-type (WT) HIV-1 molecular clone or 6A2T mutant that is defective in PIP2 binding due to mutations in the N-terminal highly basic region in the Gag MA domain. Then, the cells were cultured for 2 days, and analyzed for Gag signal by immunofluorescence. In the upper panel, the average size of Gag puncta in each Gag+ cell is summarized (20 cells for each group). In the lower panel, the number of >0.25 μm Gag puncta in each Gag+ cell is summarized (20 cells for each group). The Gag signal larger than approximately 0.03 μm was considered puncta. n.s., not significant. *p < 0.05. (B) The parental or stable M-Sec-expressing 293 cells were transfected with the indicated HIV-1 molecular clone, cultured for 2 days, and analyzed for their expression level of Gag (p55) by western blotting. β-actin blot is the loading control. (PDF) [file ppat.1013717.s019.pdf]

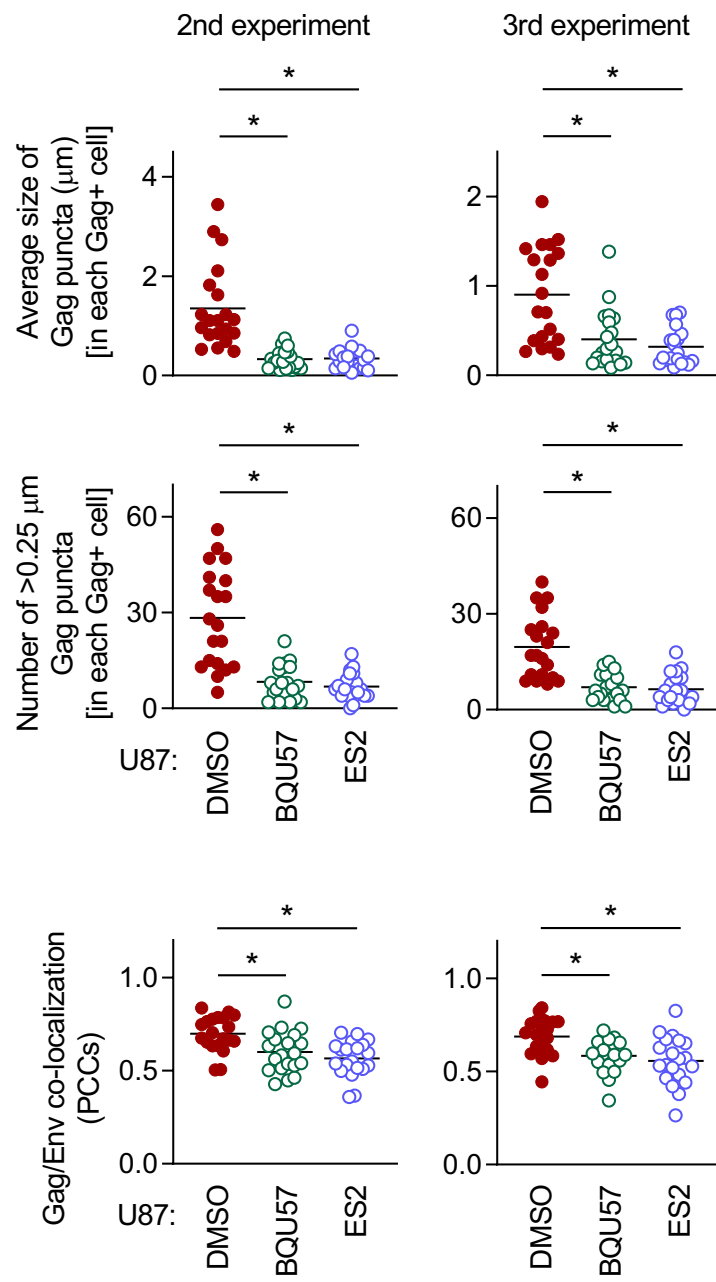

S20 Fig

Supplement: S20 Fig — (PDF) [file ppat.1013717.s020.pdf]

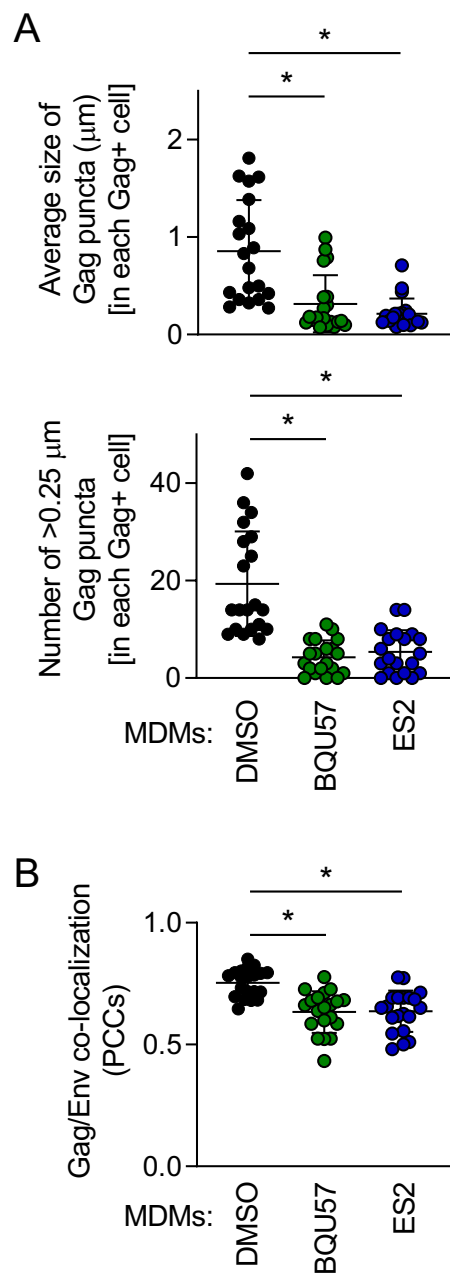

S21 Fig

Supplement: S21 Fig — (A, B) Peripheral blood monocyte-derived macrophages (MDMs) were pretreated with DMSO, 10 μM BQU57 or 10 μM ES2 for 2 days, and infected with HIV-1. Then, the cells were cultured for 2 days in the presence of DMSO, 10 μM BQU57 or 10 μM ES2, and analyzed for Gag and Env by immunofluorescence. In A, the average size of Gag puncta or the number of >0.25 μm Gag puncta in each Gag+ cell is summarized (20 cells for each group). In B, the co-localization of Gag and Env was quantified as Pearson’s correlation coefficients (PCCs) (20 cells for each group). *p < 0.05. Data shown are a representative of three independent experiments. (PDF) [file ppat.1013717.s021.pdf]

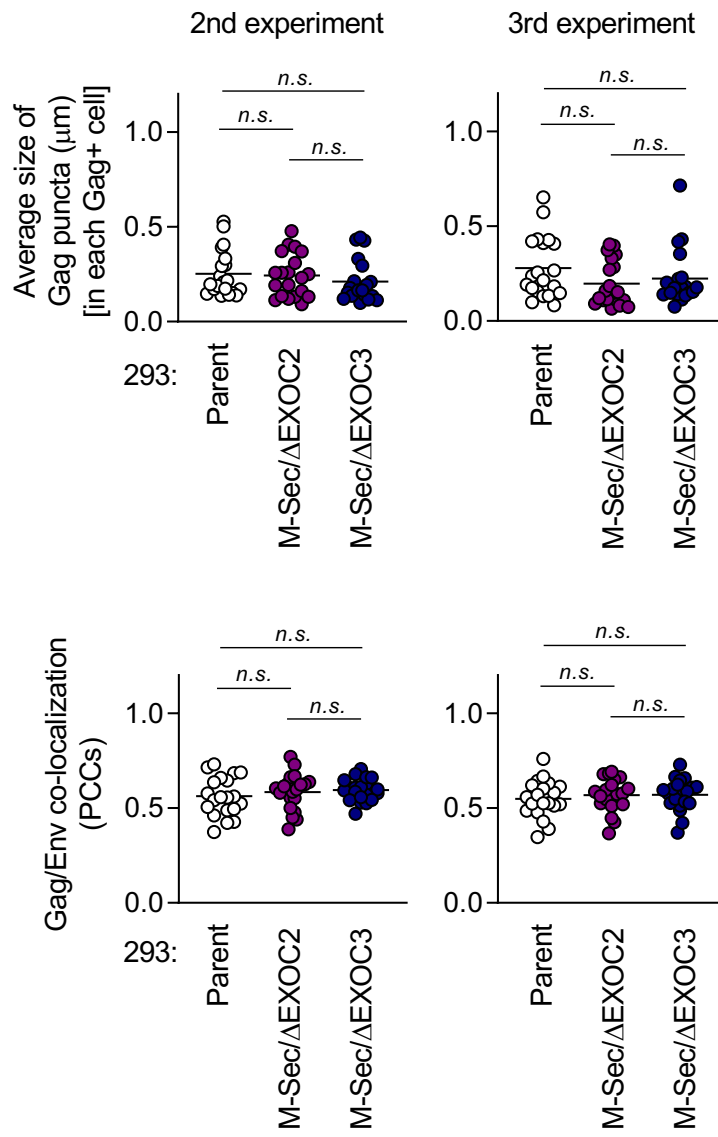

S22 Fig

Supplement: S22 Fig — (PDF) [file ppat.1013717.s022.pdf]

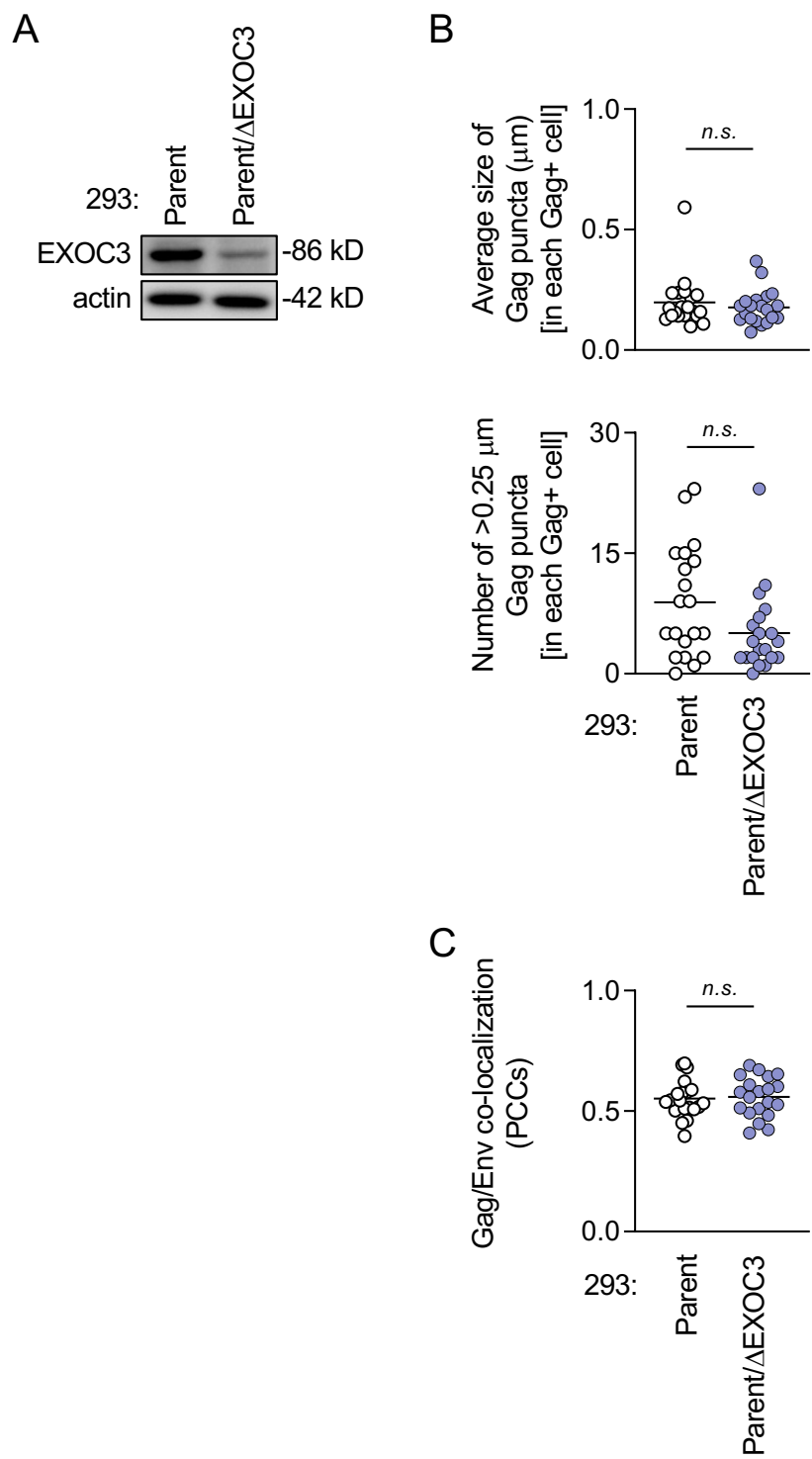

S23 Fig

Supplement: S23 Fig — (A) The parental 293 cells (Parent) or the EXOC3 knockdown parental 293 cells (Parent/ΔEXOC3) were analyzed for their expression of EXOC3 by western blotting. β- actin is the loading control. (B, C) The parental 293 cells (Parent) or the EXOC3 knockdown parental 293 cells (Parent/ΔEXOC3) were transfected with the wild-type HIV-1 molecular clone, cultured for 2 days, and analyzed for Env and Gag by immunofluorescence. In the upper panel of B, the average size of Gag puncta in each Gag+ cell is summarized (20 cells per group). In the lower panel of B, the number of >0.25 μm Gag puncta in each Gag+ cell is summarized (20 cells per group). The Gag signal larger than approximately 0.03 μm was considered puncta. In C, the co-localization of Gag and Env was quantified as Pearson’s correlation coefficients (PCCs) (20 cells per group). n.s., not significant. (PDF) [file ppat.1013717.s023.pdf]

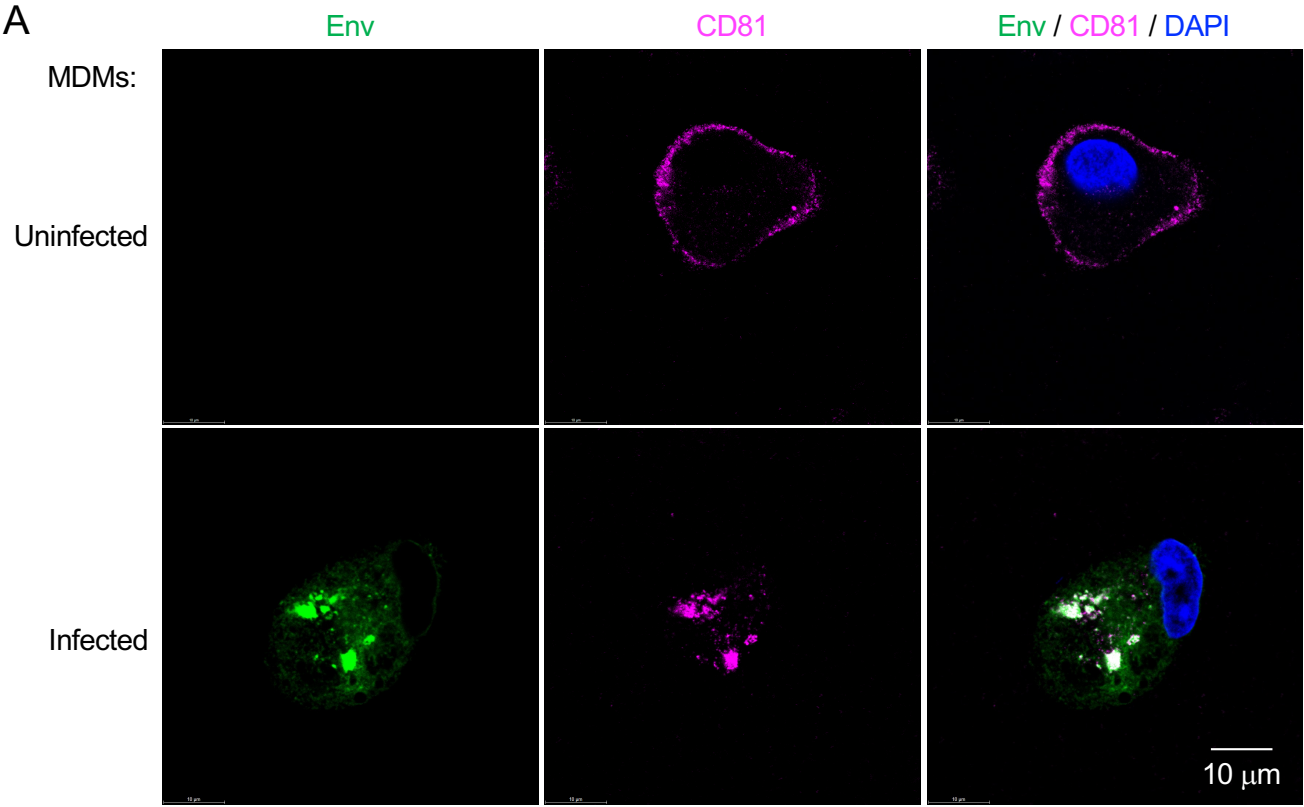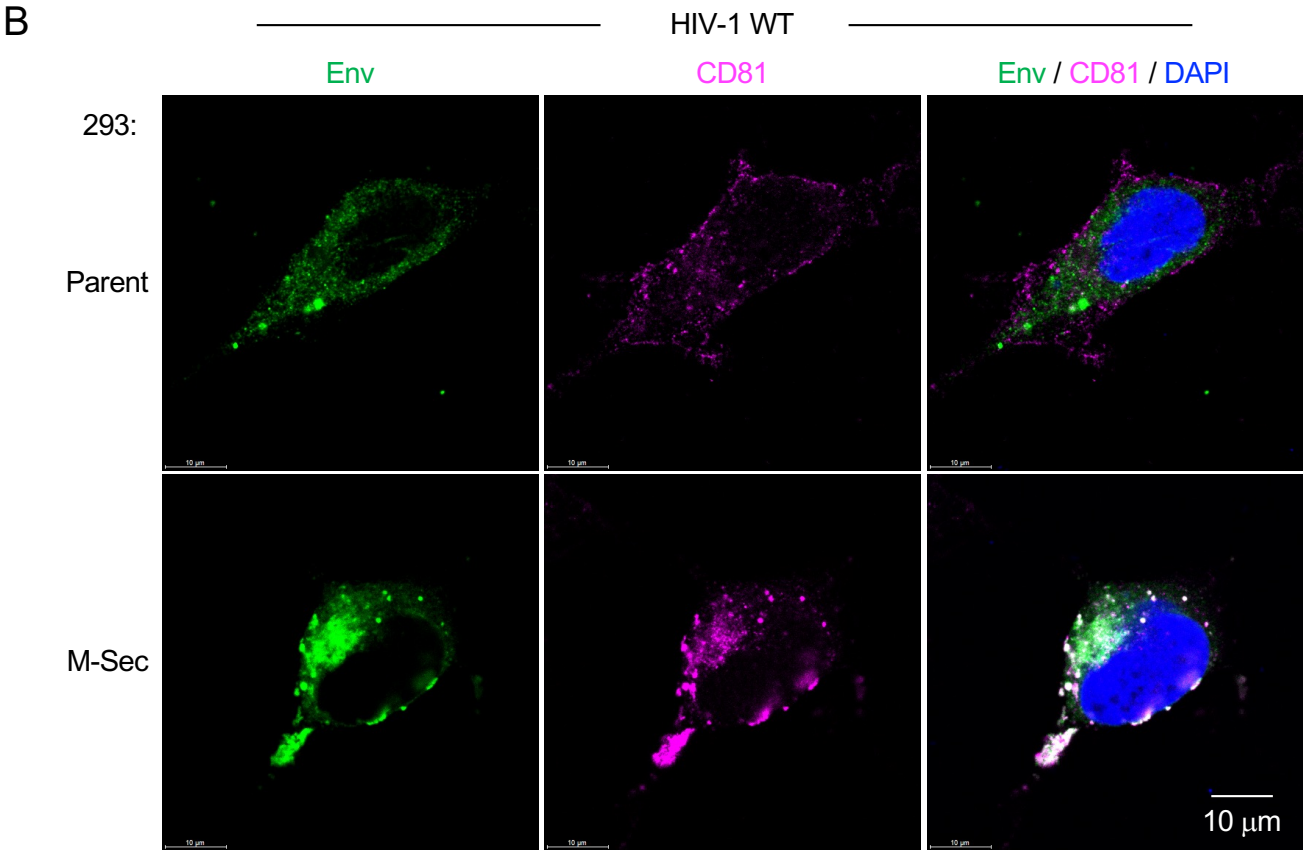

S24 Fig

Supplement: S24 Fig — (A) Peripheral blood monocyte-derived macrophages (MDMs) were left uninfected or infected with HIV-1, cultured for 2 days, and analyzed for Env (green) and CD81 (magenta) by immunofluorescence. The nuclei were stained with DAPI (blue). Scale bar: 10 μm. (B) The parental or stable M-Sec-expressing 293 cells were transfected with the wild-type HIV-1 molecular clone, cultured for 2 days, and analyzed as in A. (PDF) [file ppat.1013717.s024.pdf]

Fig. 1A:

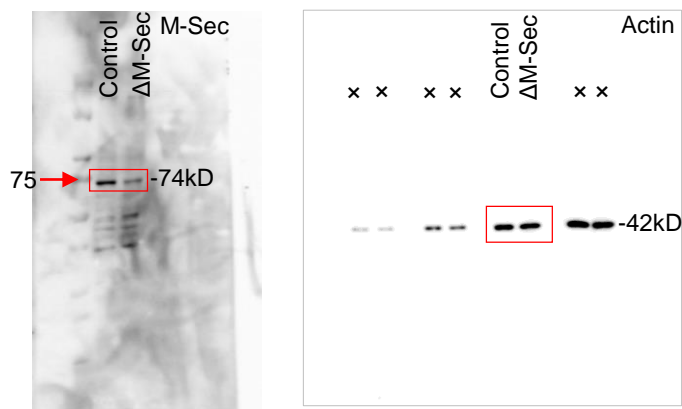

Fig. 2A:

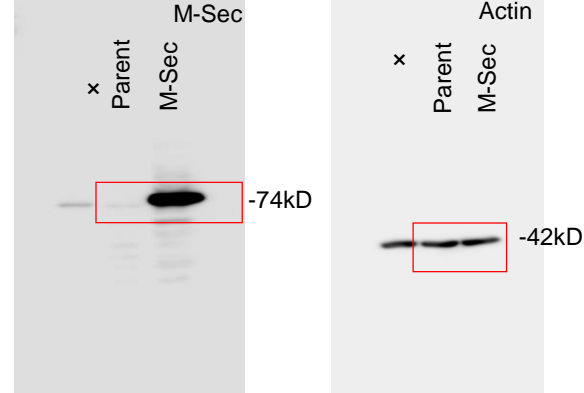

Fig. 2D:

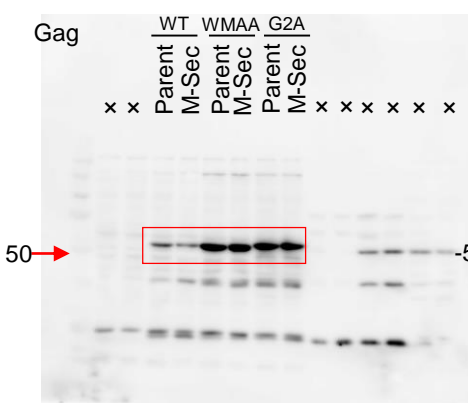

Fig.3C:

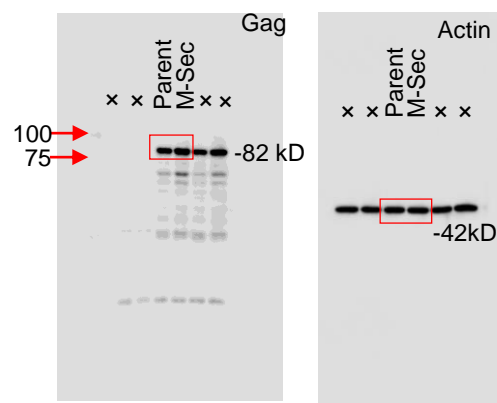

Fig.6A

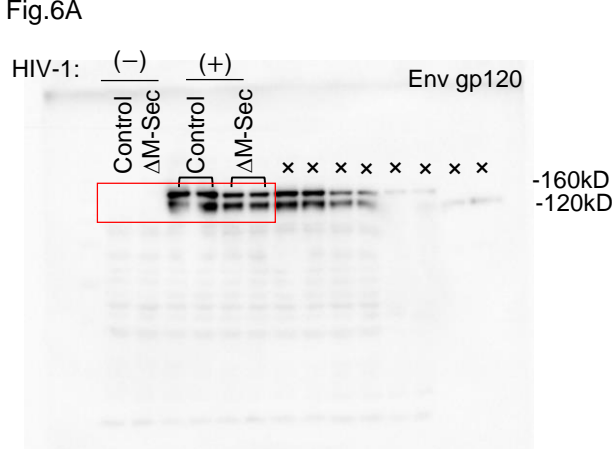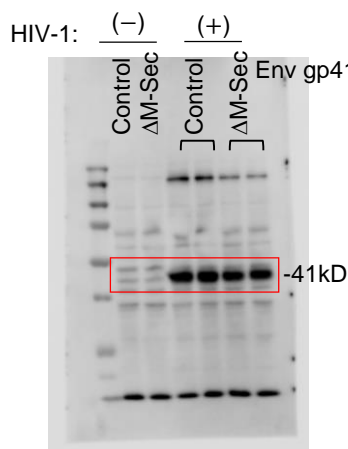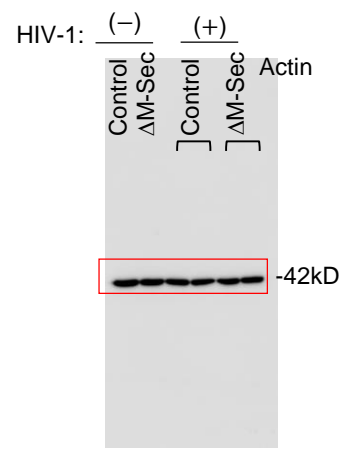

Fig.6B

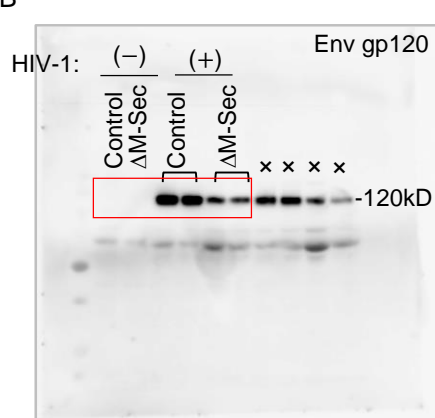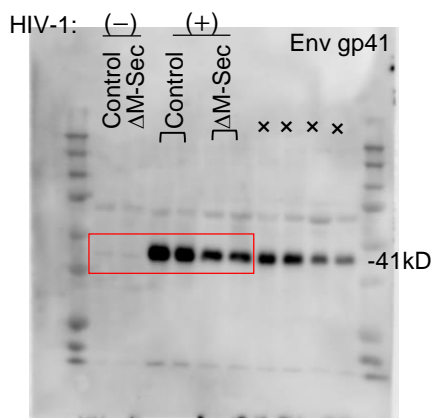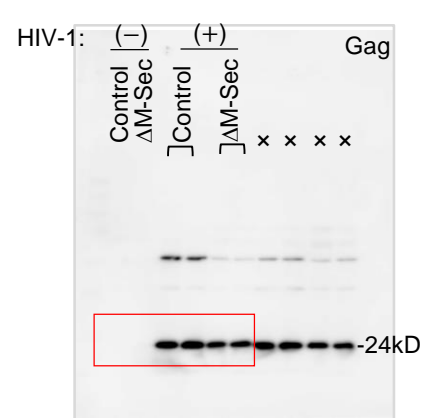

Fig. 7A

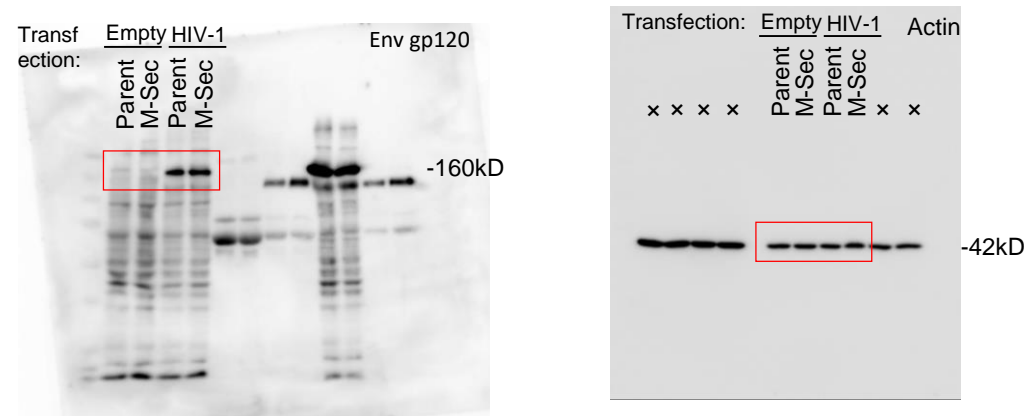

Fig. 7B

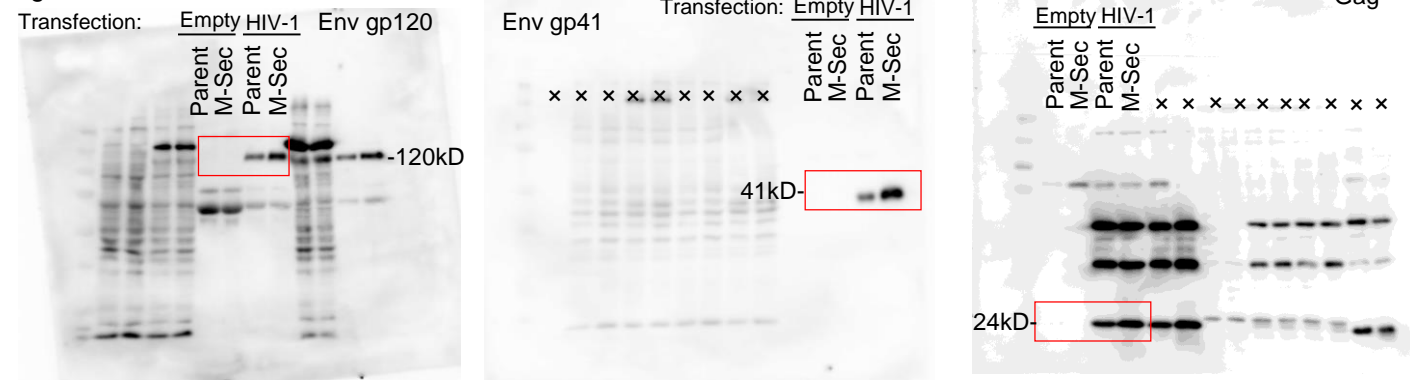

Fig.9A- Upper figure

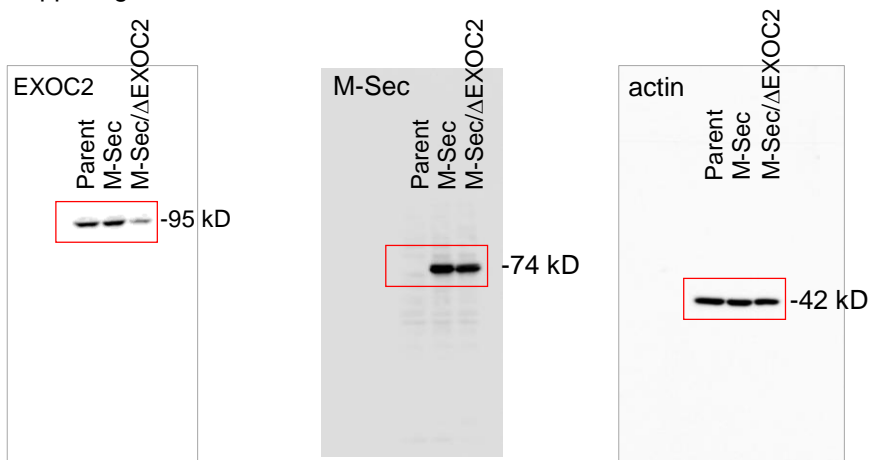

Fig.9A- lower figure

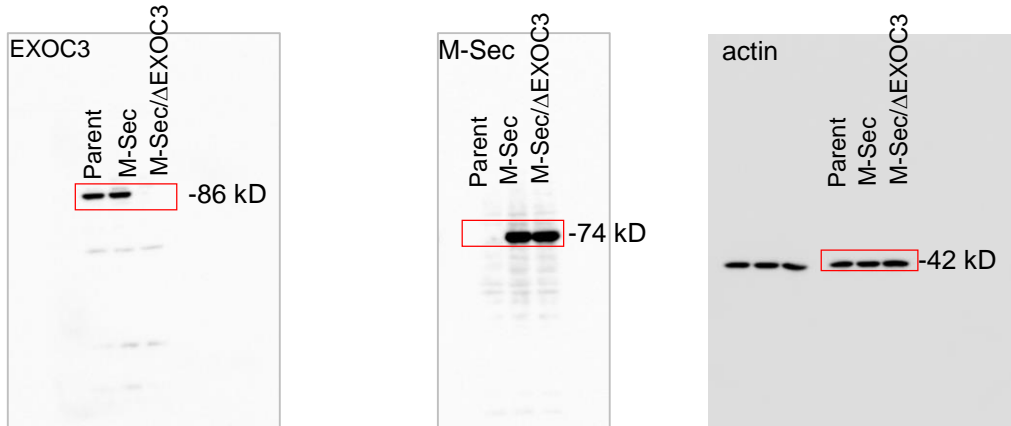

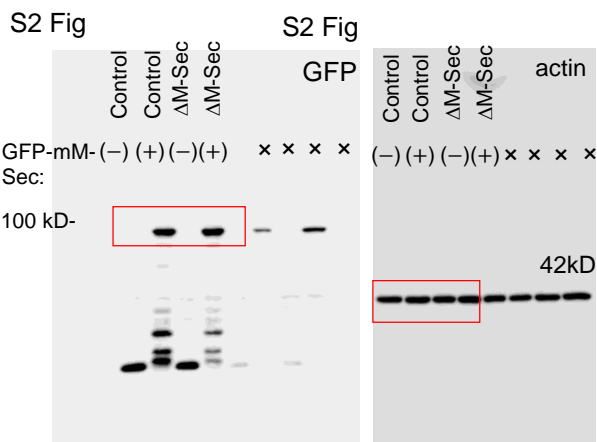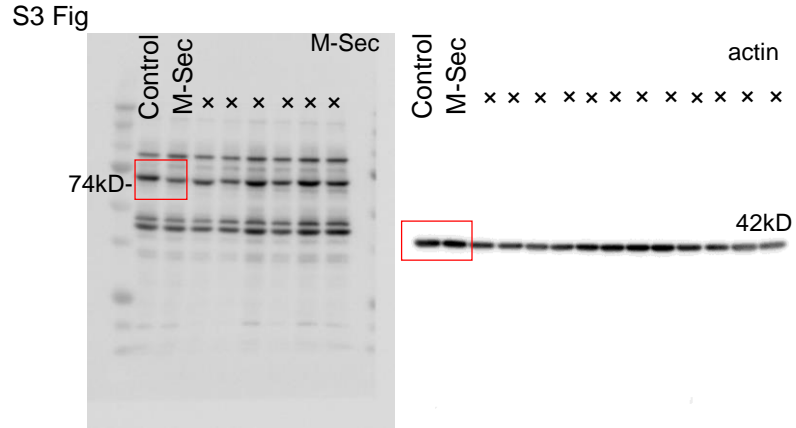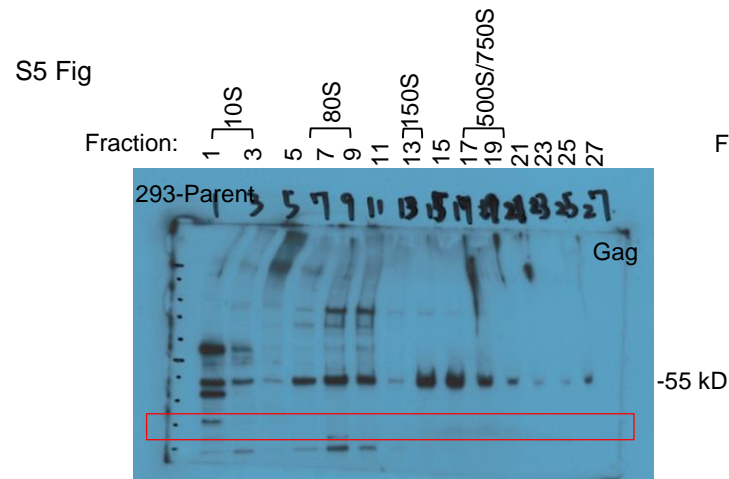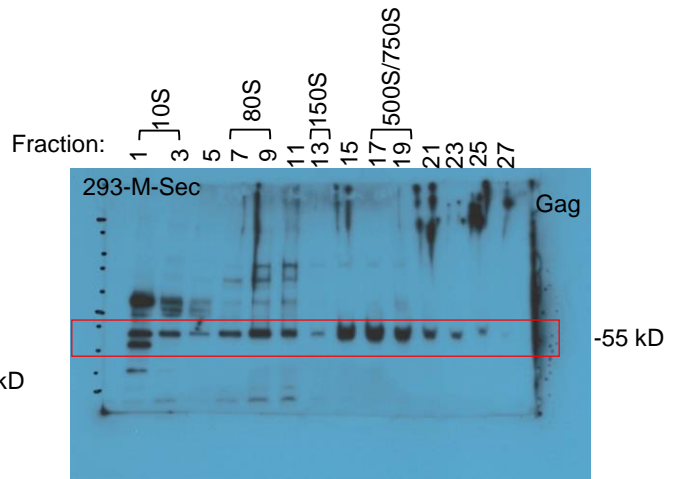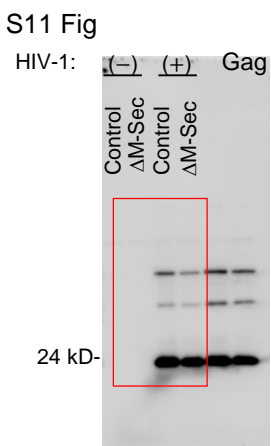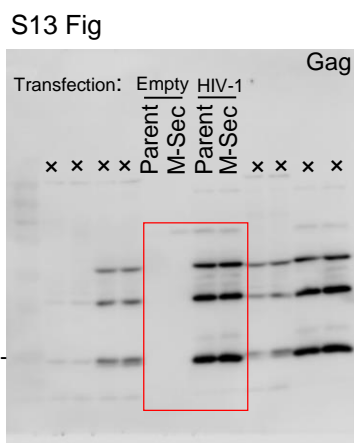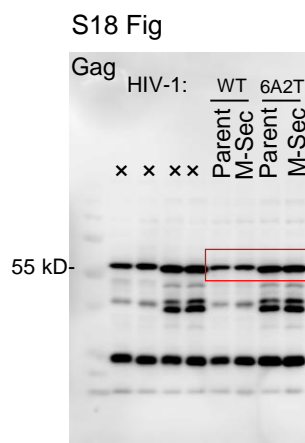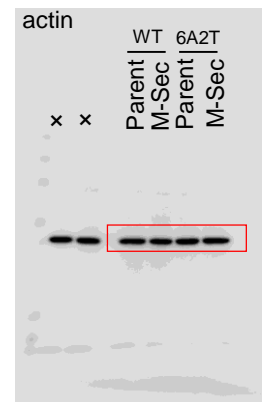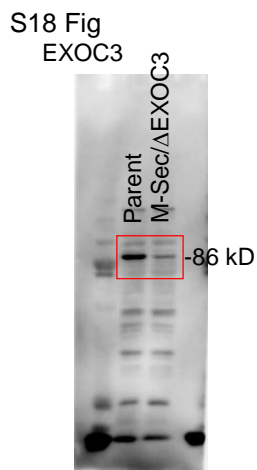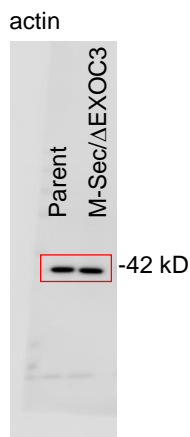

Supplement: S1 Raw images — (ZIP) [file ppat.1013717.s025.zip › S1_raw-images.pdf]
