# Supplementary material for: M-Sec promotes the production of infectious HIV-1 virus through the exocyst complex in macrophages
Source: PLoS Pathog. 2026 Jun 1;22(6):e1013717. doi: 10.1371/journal.ppat.1013717 (PMC13235924; doi:10.1371/journal.ppat.1013717)
Supplement: Attachment — Submitted filename: Reply.pdf [file ppat.1013717.s027.pdf]

Reviewer #1

Part I - Summary

Reem M. Mahmoud et al study how the cellular protein M-Sec regulates late steps of the HIV 1 life cycle and contributes to the production of infectious virions. Using knockdown in U87 cells, primary macrophages treated with the small-molecule M Sec inhibitor NPD3064, and M Sec overexpression in 293 cells, the authors show that M Sec promotes the formation and accumulation of HIV 1 Gag puncta, enhances Gag/Env co-localization, and increases Env incorporation into released particles without changing overall Env expression. There are modest changes in particle production but clear differences in infectivity, with M Sec–deficient cells producing less infectious virus and M Sec–overexpressing cells producing more infectious virus at equal RT-normalized input. Mechanistically, M Sec–dependent Gag puncta accumulation requires PIP2, the small GTPase Ral, and the exocyst complex (notably EXOC2 and EXOC3), suggesting that M Sec uses the same PIP2–Ral–exocyst axis previously implicated in tunneling nanotube formation to reorganize membranes and vesicular compartments that favor efficient Env incorporation into HIV 1 particles. The study is original and well executed. The authors propose that M Sec supports HIV 1 transmission through coordinated effects on cell structure and virion production.

Part II - Major Issues

Comment-1. Experiments with primary macrophages are limited to a pharmacological inhibitor (NPD3064) effect on Gag puncta (S1). Given M-Sec is high in macrophages and low in CD4+ T cells, showing M-Sec knockdown (siRNA/ASO) in primary macrophages with effects on Env incorporation and infectivity would substantially strengthen the biological relevance. This should be at least further discussed.

Reply

According to the comment, we performed the transient knockdown experiment by employing the protocol with which we reported an efficient knockdown of apolipoprotein E in MDMs (PLoS Pathog 14 (11), e1007372, 2018) and the siRNA with which we reported an efficient knockdown of M-Sec in U87 cells (Retrovirology 17 (1), 20, 2020). As shown in S2 Fig, the knockdown of M-Sec was partial but detectable (S2C Fig), and the total number of Gag puncta in each Gag+ M-Sec knockdown cell was smaller than that in each Gag+ control cell (S2D Fig). Although more efficient knockdown is necessary for further analyses including viral infectivity assay, we added the following sentences to explain the result of Gag puncta formation and experimental procedures (underlined):

[Page 6, lines 16 - 18 (Results)]

The knockdown of M-Sec using siRNA was partial but detectable (S2C Fig), and the total number of Gag puncta in each Gag$^+$ M-Sec knockdown MDM was smaller than that in each Gag$^+$ control MDM (S2D Fig).

[Page 17, lines 24 - 27 (Materials and methods)]

In a selected experiment, the knockdown of M-Sec was performed using siRNA (Dharmacon) and RNAiMAX (Invitrogen), as described previously [47]. siRNAs used were non-targeting siRNA (pool #2; D-001206-14) and M-Sec-specific siRNA (#4; D-012267-17) [7].

[The legend of S2 Fig]

(C, D) In C, MDMs were transfected with the control siRNA or M-Sec-specific siRNA, cultured for 4 days, and analyzed for their expression level of M-Sec by western blotting. β-actin blot is the loading control. In D, MDMs were transfected with the control siRNA or M-Sec-specific siRNA, and cultured for 2 days. Then, the cells were infected with HIV-1, cultured for 2 days, and analyzed for Gag by immunofluorescence. The total number of Gag puncta (>0.03 μm) in each Gag$^+$ cell is summarized (20 cells for each group). Data shown are a representative of three independent experiments.

Comment-2. Rescue of the U87 knockdown phenotype with shRNA-resistant M-Sec would address specificity.

Reply

According to the comment, we performed an additional experiment (the exogenous expression of mouse M-Sec in the human M-Sec knockdown U87 cells), and confirmed the rescue of Gag puncta formation (S1 Fig). To explain the result and experimental procedures, we added the following sentences and words (underlined):

[Page 6, lines 9 - 13 (Results)]

When quantified, the average size of the Gag puncta (Fig 1C, upper) or the number of >0.25 μm Gag puncta (Fig 1C, lower) in each Gag$^+$ ΔM-Sec cell was smaller than that in each Gag$^+$ control cell. Such change was rescued by the exogenous expression of mouse M-Sec in the ΔM-Sec cells (S1 Fig), excluding the possibility of off-target effects.

[Page 17, lines 15 - 17 (Materials and methods)]

In a selected experiment, U87 cells were transfected with a mouse M-Sec expression plasmid [10] using Lipofectamine 3000 (Invitrogen) [11].

[Page 20, lines 1 - 6 (Materials and methods)]

The antibodies used were as follows: anti-M-Sec (F-6; Santa Cruz Biotechnology), anti-p24 Gag (#65-004; BioAcademia), anti-gp120 Env (KD247; provided by S. Matsushita, Kumamoto University, Japan), anti-gp41 Env (2F5; obtained through BEI Resources,

NIAID, NIH), anti-EXOC2 (#12751-1-AP; Proteintech), anti-EXOC3 (#14703-1-AP; Proteintech), anti-GFP (B-2; Santa Cruz Biotechnology), and anti-β-actin (EPR16769; Abcam).

[The legend of S1 Fig]

(A, B) The control or stable M-Sec knockdown (ΔM-Sec) U87 cells were transfected with the empty vector ("−") or the GFP-fused mouse M-Sec (GFP-mM-Sec) expression plasmid ("+"), and cultured for 3 days. In A, the cells were analyzed for their expression level of GFP-mM-Sec by western blotting. β-actin blot is the loading control. In B, the cells were infected with HIV-1, cultured for 2 days, and analyzed for Gag by immunofluorescence. The number of >0.25 μm Gag puncta in each Gag^{+} cell is summarized (20 cells for each group). *n.s.*, not significant. $^{*}p < 0.05$.

Comment-3. Infectivity is normalized to RT activity; RT can vary with maturation. Is it possible to include normalization to p24 (ELISA)?

Reply

According to the comment, we performed additional experiments and confirmed that there was no obvious difference in viral production between RT activity assay and p24 Gag ELISA (S7 Fig and S9 Fig for U87 cells and 293 cells, respectively). To explain the results and experimental procedures, we added the following sentences and words (underlined):

[Page 9, lines 9 - 12 (Results)]

The ΔM-Sec cells produced slightly lesser amount of virus than the control because the reverse transcriptase (RT) activity in the supernatants of the ΔM-Sec cells was slightly weaker than that of the control cells (Fig 6D), which was confirmed by p24 Gag ELISA (S7 Fig).

[Page 9, lines 21 - 24 (Results)]

The M-Sec-expressing cells produced slightly higher amount of virus than the parental cells (Fig 7D, S9 Fig), and those viruses showed stronger infectivity than those by the parental cells (Fig 7E).

[Page 22, lines 12 and 13 (Materials and methods)]

The viral production was also monitored by p24 Gag ELISA (Rimco, Okinawa, Japan).

[The legend of S7 Fig]

The control or stable M-Sec knockdown (ΔM-Sec) U87 cells were infected with HIV-1, and cultured for 2 days. The supernatants were collected, and analyzed for the activity of reverse transcriptase (RT) by qPCR (n=3, left), or p24 Gag by ELISA (n=3, right). $^{*}p < 0.05$.

[The legend of S9 Fig]

The parental or stable M-Sec-expressing 293 cells were transfected with the empty plasmid or HIV-1 molecular clone, and cultured for 2 days. The supernatants were collected, and analyzed for the activity of reverse transcriptase (RT) by qPCR (n=3, left), or p24 Gag by ELISA (n=3, right). $^{*}p < 0.05$.

Comment-4. Assessing Gag processing/maturation in virions will allow to exclude M-Sec effects on protease-mediated maturation that could confound infectivity.

Reply

According to the comment, we showed the results in S6 Fig and S8 Fig. The Gag processing was almost unchanged by the M-Sec knockdown in U87 cells (S6 Fig) or the exogenous M-Sec expression in 293 cells (S8 Fig). To explain the results and experimental procedures, we added the following sentences (underlined):

[Page 9, lines 6 - 9 (Results)]

However, the virus-like particles produced by the ΔM-Sec cells contained a lesser amount of gp120 and gp41 than those by the control cells (Fig 6B), even after the normalization to mature Gag (p24) in the virus-like particles (Fig 6C). The processing of Gag was almost unchanged (S6 Fig).

[Page 9, lines 18 - 21 (Results)]

However, the virus-like particles produced by the M-Sec-expressing cells contained higher amount of gp120 and gp41 than those by the parental cells (Fig 7B), even after the normalization to p24 Gag in the virus-like particles (Fig 7C). The processing of Gag was almost unchanged (S8 Fig).

[The legend of S6 Fig]

(A, B) The control or stable M-Sec knockdown (ΔM-Sec) U87 cells were left uninfected or infected with HIV-1, and cultured for 2 days. The virus-like particles in the supernatants were collected by centrifugation, and analyzed for their amount of p55 Gag, p41 Gag, and p24 Gag by western blotting. In B, the percentage of each Gag protein to the total Gag proteins is summarized (n=3).

[The legend of S8 Fig]

(A, B) The parental or stable M-Sec-expressing 293 cells were transfected with the empty plasmid or HIV-1 molecular clone, and cultured for 2 days. The virus-like particles in the supernatants were collected by centrifugation, and analyzed for their amount of p55 Gag, p41 Gag, and p24 Gag by western blotting. In B, the percentage of each Gag protein to the total Gag proteins is summarized (n=3).

Comment-5. Can the authors provide quantification (densitometry, replicates) for EXOC2/EXOC3 knockdown efficiency and show that basal Gag puncta/Env colocalization in parental cells are affected similarly by exocyst knockdown?

Reply

According to the comment, we added the quantification for EXOC2/EXOC3 knockdown efficiency (Fig 9A). Also, we performed an additional experiment and found that there was no further reduction in the Gag puncta formation and Gag/Env co-localization in the EXOC3 knockdown parental 293 cells (S15 Fig). To explain the results and experimental procedures, we added the following words and sentences (underlined):

[The legend of Fig 9A]

(A) In the upper blots, the parental (Parent), M-Sec-expressing (M-Sec), or stable EXOC2 knockdown M-Sec-expressing (M-Sec/ΔEXOC2) 293 cells were analyzed for their expression of EXOC2 or M-Sec by western blotting. β-actin blot is the loading control. In the graph, the density of EXOC2 band of the M-Sec/ΔEXOC2 293 cells is represented as a percentage relative to that of the M-Sec 293 cells (n=3). In the lower blots, the stable EXOC3 knockdown M-Sec-expressing (M-Sec/ΔEXOC3) 293 cells were analyzed for their expression of EXOC3. In the graph, the density of EXOC3 band of the M-Sec/ΔEXOC3 293 cells is represented as a percentage relative to that of the M-Sec 293 cells (n=3). $^{*}p < 0.05$.

[Page 11, line 27 - page 12, line 2 (Results)]

There was no further reduction in the Gag puncta formation and Gag/Env co-localization in the EXOC3 knockdown parental cells (S15 Fig).

[The legend of S15 Fig]

(A) The parental 293 cells (Parent) or the EXOC3 knockdown parental 293 cells (Parent/ΔEXOC3) were analyzed for their expression of EXOC3 by western blotting. β-actin is the loading control.

(B, C) The parental 293 cells (Parent) or the EXOC3 knockdown parental 293 cells (Parent/ΔEXOC3) were transfected with the wild-type HIV-1 molecular clone, cultured for 2 days, and analyzed for Env and Gag by immunofluorescence. In the upper panel of B, the average size of Gag puncta in each Gag^{+} cell is summarized (20 cells per group). In the lower panel of B, the number of >0.25 µm Gag puncta in each Gag^{+} cell is summarized (20 cells per group). The Gag signal larger than approximately 0.03 µm was considered puncta. In C, the co-localization of Gag and Env was quantified as Pearson's correlation coefficients (PCCs) (20 cells per group). *n.s.*, not significant.

Comment-6. It would be worth testing Gag MA basic patch mutants (defective in PIP2

binding) to determine whether M-Sec's effect requires Gag–PIP2 interactions. This could be at least discussed.

Reply

According to the comment, we performed an additional experiment using the 6A2T mutant that is defective in PIP2 binding due to mutations in the N-terminal highly basic region in the Gag MA domain (ref. 23). As shown in S13 Fig, we confirmed that the effect of M-Sec requires Gag - PIP2 binding. To explain the results and experimental procedures, we added the following sentences and words (underlined):

[Page 10, lines 19 - 26 (Results)]

To confirm the requirement of PIP2 for the M-Sec-mediated accumulation of Gag puncta, we depleted PIP2 in the M-Sec-expressing 293 cells by the over-expression of inositol polyphosphate 5-phosphatase type IV (5ptaseIV) [21,22]. The wild-type (WT), 5ptaseIV, but not the defective mutant lacking the 5-phosphatase domain (Δ1), reduced the average size of the Gag puncta (S7 Fig, upper) or the number of >0.25 μm Gag puncta (S7 Fig, lower) in each Gag^+ M-Sec-expressing cell. Consistent with this result, M-Sec failed to induce the accumulation of puncta when we used the 6A2T Gag mutant (S13 Fig), which is defective in PIP2 binding [23].

[Page 19, lines 10 - 12 (Materials and methods)]

The molecular clones used were the wild-type JRFL strain, or the wild-type NL43 strain and its mutants WMAA, G2A, or 6A2T [12,13,23].

[The legend of S13 Fig]

(A) The parental or stable M-Sec-expressing 293 cells were transfected with the wild-type (WT) HIV-1 molecular clone or 6A2T mutant that is defective in PIP2 binding due to mutations in the N-terminal highly basic region in the Gag MA domain. Then, the cells were cultured for 2 days, and analyzed for Gag signal by immunofluorescence. In the upper panel, the average size of Gag puncta in each Gag^+ cell is summarized (20 cells for each group). In the lower panel, the number of >0.25 μm Gag puncta in each Gag^+ cell is summarized (20 cells for each group). The Gag signal larger than approximately 0.03 μm was considered puncta. *n.s.*, not significant. $^*p < 0.05$.

(B) The parental or stable M-Sec-expressing 293 cells were transfected with the indicated HIV-1 molecular clone, cultured for 2 days, and analyzed for their expression level of Gag (p55) by western blotting. β-actin blot is the loading control.

Comment-7-1. Image quantifications often use 10–15 cells per group, and only one or two cells are shown in the figures. What are the number of independent biological replicates? It may be worth showing lower magnification images with more cells.

Reply

According to the comment, we showed the results with 20 cells for each group in Figs 1C, 2C, 3B, 4B, 5B, 5C, 8B, 8C, 9C, 9D, and S1B, S2B, S2D, S5, S12, S13, S14, S15B, S15C). The data were obtained in the same experiments and a representative of three independent experiments. To clarify this point, we added the following sentence to the figure legend (underlined): Data shown are a representative of three independent experiments.

Comment-7-2. It may be worth showing lower magnification images with more cells.

Reply

We showed lower magnification images of U87 cells in Fig 1B (left panels), but a further lower magnification with more cells may not be enough to identify the puncta formation of Gag in our system.

Reviewer #2

Part I - Summary

HIV-1 infection of target cells can occur via cell-associated transmission modes or by cell-free particles. Previous work by the Suzu lab established that the M-Sec protein is involved in cell-associated transmission of HTLV and HIV-1. In the present study by Mahmoud and colleagues, the group analyzes the impact of M-Sec and production and infectivity of cell free HIV-1 particles. To this end, endogenous M-Sec expression is either reduced or M-Sec is ectopically overexpressed in virus cells that produce HIV-1 particles following transfection with proviral DNA or infection with HIV-1. The authors describe that reduced M-Sec expression reduces the accumulation of HIV-1 in intracellular puncta, while overexpression promotes the intracellular accumulation of Gag. Similar approaches also document a subtle effect of M-Sec on the recruitment of the viral glycoprotein Env to these intracellular accumulations and incorporation of Env into viral particles. Consistently, the presence of M-Sec has a moderate effect on the infectivity of these particles. Pharmacological inhibition of the Ral GTPase or EXOC7 abrogates the effects of M-Sec depletion or overexpression. The study addresses a relevant topic and presents interesting new data.

Part II - Major Issues

Comment-1-1. Most of the results in this study are deduced from microscopy analyses in which a few individual cells were analyzed (typically around 15). Although I note that this is how previous papers from the group have been published, this does not seem to be sufficient to draw meaningful conclusions. It is also not clear if these 15 cells were acquired in the same experiments or collected in different experiments. Considering that this is a straightforward analysis and cells to be analyzed are not a rare resource, analyzing at least three independent experiments with at least 20 cells per condition would seem a minimum.

Reply

According to the comment, we showed the results with 20 cells for each group in Figs 1C, 2C, 3B, 4B, 5B, 5C, 8B, 8C, 9C, 9D, and S1B, S2B, S2D, S5, S12, S13, S14, S15B, S15C). The data were obtained in the same experiments and a representative of three independent experiments. To clarify this point, we added the following sentence to the figure legend (underlined): Data shown are a representative of three independent experiments.

Comment-1-2. Also, the authors refer to previous papers for quality controls for the

functional impact of M-Sec depletion on particle production. Since depletion is partial and stable cell lines may phenotypically adapt to the loss M-Sec expression, including this control as standard control in each experiment would be helpful.

Reply

We appreciate the comment. To exclude the possibility that U87 cells adapt to the loss of M-Sec expression, we performed an additional experiment (the exogenous expression of mouse M-Sec in the human M-Sec knockdown U87 cells), and confirmed the rescue of Gag puncta formation (S1 Fig). To explain the results and experimental procedures, we added the following sentences and words (underlined):

[Page 6, lines 9 - 13 (Results)]

When quantified, the average size of the Gag puncta (Fig 1C, upper) or the number of >0.25 μm Gag puncta (Fig 1C, lower) in each Gag$^+$ ΔM-Sec cell was smaller than that in each Gag$^+$ control cell. Such change was rescued by the exogenous expression of mouse M-Sec in the ΔM-Sec cells (S1 Fig), excluding the possibility of off-target effects.

[Page 17, lines 15 - 17 (Materials and methods)]

In a selected experiment, U87 cells were transfected with a mouse M-Sec expression plasmid [10] using Lipofectamine 3000 (Invitrogen) [11].

[Page 20, lines 1 - 6 (Materials and methods)]

The antibodies used were as follows: anti-M-Sec (F-6; Santa Cruz Biotechnology), anti-p24 Gag (#65-004; BioAcademia), anti-gp120 Env (KD247; provided by S. Matsushita, Kumamoto University, Japan), anti-gp41 Env (2F5; obtained through BEI Resources, NIAID, NIH), anti-EXOC2 (#12751-1-AP; Proteintech), anti-EXOC3 (#14703-1-AP; Proteintech), anti-GFP (B-2; Santa Cruz Biotechnology), and anti-β-actin (EPR16769; Abcam).

[The legend of S1 Fig]

(A, B) The control or stable M-Sec knockdown (ΔM-Sec) U87 cells were transfected with the empty vector ("−") or the GFP-fused mouse M-Sec (GFP-mM-Sec) expression plasmid ("+"), and cultured for 3 days. In A, the cells were analyzed for their expression level of GFP-mM-Sec by western blotting. β-actin blot is the loading control. In B, the cells were infected with HIV-1, cultured for 2 days, and analyzed for Gag by immunofluorescence. The number of >0.25 μm Gag puncta in each Gag$^+$ cell is summarized (20 cells for each group). *n.s.*, not significant. $^*p < 0.05$.

Comment-2-1. The differences observed upon M-Sec depletion or overexpression in Gag relocalization, Env recruitment and virion incorporation, and particle infectivity are subtle (factor 2 or less). This does not mean that this is not an important phenomenon but it

would need to be acknowledged more clearly as a very subtle phenotype. Maybe assessing the role of M-Sec over several rounds of replication would help to illustrate the relevance of the effect? Does the magnitude of infectivity alteration differ between Env variants with different tropism, tendency for shedding or other parameters?

Reply

As pointed out, the difference in viral infectivity induced by the M-Sec knockdown or over-expression was subtle. The Nef-deficient virus is well known to exhibit a weak infectivity. As shown below, its infectivity is approximately half of that of the wild-type virus. Meanwhile, the enhancement of viral infectivity by the M-Sec over-expression in 293 cells is detectable but less than two-fold. Thus, to clarify its impact on a long-term viral replication, further study is necessary. Among parameters tested, the viral production correlated with the magnitude of infectivity alteration (Fig 6, Fig 7). To discuss these points, we added the following sentences (underlined):

[Page 15, lines 13 - 16 (Discussion)]

The difference in viral infectivity induced by the M-Sec knockdown or over-expression correlated with the difference in viral production (Fig 6, Fig 7). However, its degree was subtle. Thus, to clarify its impact on a long-term viral replication, further study is necessary.

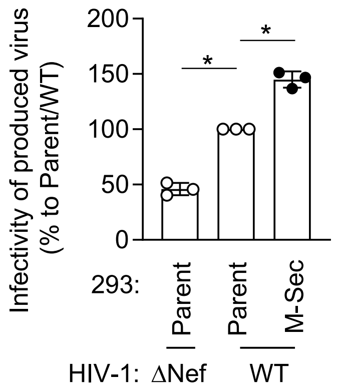

The parental or M-Sec-expressing 293 cells were transfected with the wild-type (WT) or Nef-deficient HIV-1 molecular clone, and cultured for 2 days. The supernatants were collected and analyzed for viral infectivity using TZM-bl cells as the target cells (the input: 2 nU/mL of reverse transcriptase activity). The infectivity is represented as a percentage relative to that of the parental cells transfected with WT HIV-1 (n=3). *$p < 0.05$.

Comment-2-2. Does the magnitude of infectivity alteration differ between Env variants with different tropism, tendency for shedding or other parameters?

Reply

According to the comment, we performed an additional experiment. As shown in this manuscript (the M-tropic JRFL strain) and the figure below (the T-tropic NL43 strain), the M-Sec over-expression in 293 cells promotes the production of infectious viral particles regardless of the tropism.

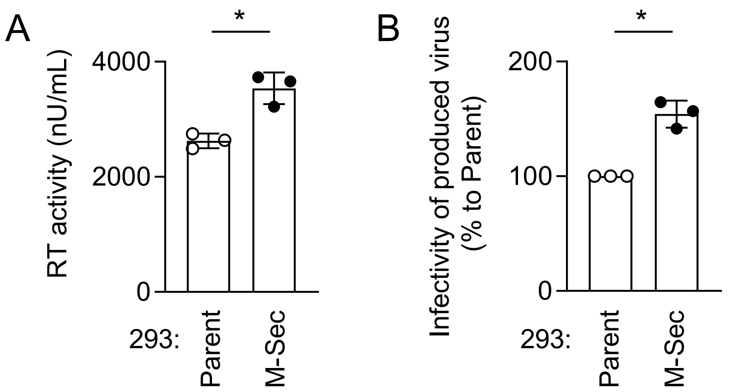

The parental or M-Sec-expressing 293 cells were transfected with the HIV-1 molecular clone (NL4-3), and cultured for 2 days. In **A**, the supernatants were collected and analyzed for the activity of reverse transcriptase (RT) by qPCR (n=3). In **B**, the supernatants were collected, and analyzed for viral infectivity using TZM-bl cells as the target cells (the viral input: 4 nU/mL RT activity). The infectivity is represented as a percentage relative to that of the parental cells (n=3). $^{*}p < 0.05$.

Comment-3. Fig. 8: data on the infectivity of particles produced from cells treated with these inhibitors should be presented.

Reply

According to the comment, we performed additional experiments. In particular, we used monocyte-derived macrophages (MDMs), more physiological HIV-1 target than U87 cells, and confirmed that both BQU57 and ES2 reduced the viral infectivity (Fig 8D), the Gag puncta formation (S14A Fig), and the Gag/Env co-localization (S14B Fig). To explain the results and experimental procedures, we added the following sentences (underlined):

[Page 11, lines 11 - 16 (Results)]

When pretreated with either BQU57 or ES2, and infected with HIV-1, the control U87 cells showed the M-Sec knockdown cell-like phenotypes, i.e., the smaller Gag puncta (Fig 8A, 8B) and the weaker Gag/Env co-localization (Fig 8A, 8C). Similar results were obtained for MDMs (S14 Fig). Moreover, viruses produced by MDMs pretreated with BQU57 or ES2 showed weaker infectivity than those produced by the DMSO-treated control MDMs (Fig 8D).

[The legend of Fig 8D]

(D) Peripheral blood monocyte-derived macrophages (MDMs) obtained from two different donors (Donor-1 and Donor-2) were pretreated with DMSO, 10 μM BQU57 or 10 μM ES2 for 2 days, and infected with HIV-1. Then, the cells were cultured for 2 days

in the presence of DMSO, 10 μM BQU57 or 10 μM ES2. The supernatants were collected, and analyzed for viral infectivity using TZM-bl cells as the target cells (the viral input: 4 nU/mL reverse transcriptase activity). The infectivity is represented as a percentage relative to that of the DMSO-treated control MDMs (n=3).

[The legend of S14 Fig]

(A, B) Peripheral blood monocyte-derived macrophages (MDMs) were pretreated with DMSO, 10 μM BQU57 or 10 μM ES2 for 2 days, and infected with HIV-1. Then, the cells were cultured for 2 days in the presence of DMSO, 10 μM BQU57 or 10 μM ES2, and analyzed for Gag and Env by immunofluorescence. In A, the average size of Gag puncta or the number of >0.25 μm Gag puncta in each Gag^{+} cell is summarized (20 cells for each group). In B, the co-localization of Gag and Env was quantified as Pearson's correlation coefficients (PCCs) (20 cells for each group). $^{*}p < 0.05$. Data shown are a representative of three independent experiments.

Reviewer #3

Part I - Summary

The authors very recently reported in PLOS Pathogens that the host protein "M-Sec promotes the accumulation of intracellular HTLV-1 Gag puncta and the incorporation of Env into viral particles". In the present study, they essentially repeated this study with HIV-1, and arrived largely at the same conclusions. Specifically, they show that the depletion of M-Sec reduces, and exogenous M-Sec increases, intracellular HIV-1 Gag puncta, and that HIV-1 Env appears to co-localize with these Gag puncta. They also show that the M-Sec-mediated accumulation of Gag puncta depends on PIP2 (as it did in their previous paper on HTLV-I) and on Ral and the exocyst complex (whose SEC6 component exhibits sequence similarity with M-Sec). Additionally, they show that M-Sec promotes Env incorporation into viral particles and their infectivity. However, these effects were small (generally less than 2-fold), and whether these occur in relevant HIV-1 target cells was not examined. Altogether, the present study appears to be somewhat confirmatory in nature, in the sense that it extends previous findings to yet another retrovirus. It is also difficult to reconcile with an earlier study by the same authors, in which M-Sec only affected HIV-1 particle production in the presence of Nef.

Part II - Major Issues

Comment-1. The authors show that M-Sec induces the accumulation of HIV-1 Gag puncta independently of other viral components (such as Nef). How can this finding be reconciled with their previously reported observation that in the absence of Nef, M-Sec had no effect on HIV-1 virus production in a spreading infection assay (Fig. 5 of ref. 7).

Reply

We appreciate the thoughtful comment. As pointed out, we previously reported that the M-Sec knockdown reduced the production of the wild-type virus, but not that of Nef-deficient virus (ref. 7). The Nef-deficient virus replicated less efficiently than the wild-type virus (ref. 7), and Nef was required for the M-Sec-mediated tunneling nanotube formation (ref. 7). Thus, we speculated that Nef played a role in the M-Sec-mediated viral production through the tunneling nanotube formation. However, the speculation might not necessarily fully explain the reduced production of the wild-type virus by the M-Sec knockdown because the M-Sec-mediated Gag puncta formation was independent of Nef, which was further confirmed by an additional experiment using the Nef-deficient HIV-1 molecular clone (please see the figure below). Thus, M-Sec may contribute to HIV-1 production in both Nef-dependent and Nef-independent manners. To discuss this point, we added the following sentences (underlined):

[Page 15, lines 4 - 12 (Discussion)]

We previously reported that the M-Sec knockdown reduced the production of the wild-type virus, but not that of Nef-deficient virus [7]. The Nef-deficient virus replicated less efficiently than the wild-type virus [7], and Nef was required for the M-Sec-mediated tunneling nanotube formation [7]. Thus, we speculated that Nef played a role in the M-Sec-mediated viral production through the tunneling nanotube formation. However, the speculation might not necessarily fully explain the reduced production of the wild-type virus by the M-Sec knockdown because the M-Sec-mediated Gag puncta formation was independent of Nef. Thus, M-Sec may contribute to HIV-1 production in both Nef-dependent and Nef-independent manners.

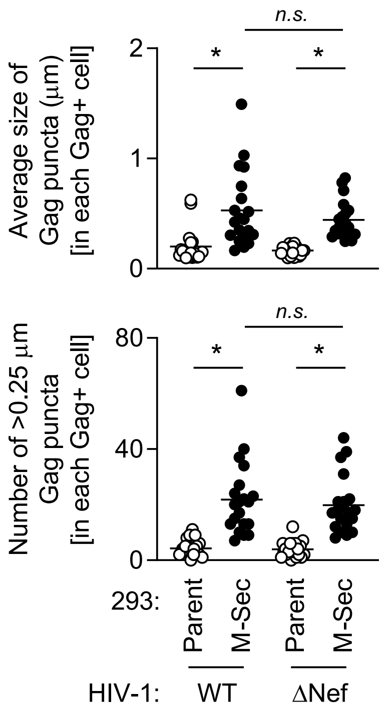

The parental or M-Sec-expressing 293 cells were transfected with the wild-type (WT) or Nef-deficient (ΔNef) HIV-1 molecular clone, cultured for 2 days, and analyzed for Gag by immunofluorescence. *Upper,* the average size of Gag puncta in each Gag+ cell is summarized (20 cells for each group). *Lower*, the number of >0.25 μm Gag puncta in each Gag+ cell is summarized (20 cells for each group). The Gag signal larger than approximately 0.03 μm was considered puncta. *n.s.*, not significant. *$p < 0.05$.

Comment-2. The M-Sec-induced "Gag puncta" often are quite large, and appear to be mostly in a peri-nuclear location and rather than at the PM. What evidence do the authors have that these "Gag puncta" are not simply non-specific Gag aggregates, rather than assembling HIV-1 virions, as the authors seem to assume?

Reply

We appreciate the comment. The M-Sec-induced large Gag puncta co-localized with Env (Fig 4, Fig 5). The accumulation of virus-like particles was often detected in M-Sec-expressing cells by an electron microscopic analysis (Fig 2E), the structures of which resembled the virus-containing compartments (VCCs) in macrophages (ref. 46). The

additional data shown in S16 Fig supported the hypothesis that the unique structures often detected in the M-Sec-expressing cells represent VCCs, although further study is necessary to prove the hypothesis. To explain the results and experimental procedures, we added the following sentences (underlined):

[Page 15, line 25 - page 16, line 4 (Discussion)]

Consistent with this idea, the accumulation of virus-like particles was often detected in the M-Sec-expressing cells by an electron microscopic analysis (Fig 2E), the structures of which resemble the virus-containing compartments (VCCs) in macrophages [46]. In fact, the signal of Env in MDMs and the M-Sec-expressing 293 cells overlapped with the signal of CD81 (S16 Fig), a widely-used marker of VCCs [46]. Further study is necessary to prove the hypothesis that M-Sec plays a role in the formation of VCCs.

[Page 21, line 2 (Materials and methods)]

In a selected experiment, anti-CD81 (5A6; BioLegend) was used.

[The legend of S16 Fig]

(A) Peripheral blood monocyte-derived macrophages (MDMs) were left uninfected or infected with HIV-1, cultured for 2 days, and analyzed for Env (green) and CD81 (magenta) by immunofluorescence. The nuclei were stained with DAPI (blue). Scale bar: 10 μm.

(B) The parental or stable M-Sec-expressing 293 cells were transfected with the wild-type HIV-1 molecular clone, cultured for 2 days, and analyzed as in A.

Comment-3. The conclusions largely rely on the use of shRNAs to stably knock down host proteins such as M-Sec in a cell line. However, in the experience of this reviewer, the stable expression of shRNAs frequently and significantly impairs HIV-1 replication in a manner that has nothing to do with the depletion of the intended target. It is therefore imperative that the authors perform rescue experiments.

Reply

According to the comment, we performed an additional experiment (the exogenous expression of mouse M-Sec in the human M-Sec knockdown U87 cells), and confirmed the rescue of Gag puncta formation (S1 Fig). To explain the result and experimental procedures, we added the following sentences and words (underlined):

[Page 6, lines 9 - 13 (Results)]

When quantified, the average size of the Gag puncta (Fig 1C, upper) or the number of >0.25 μm Gag puncta (Fig 1C, lower) in each Gag^+ ΔM-Sec cell was smaller than that in each Gag^+ control cell. Such change was rescued by the exogenous expression of mouse M-Sec in the ΔM-Sec cells (S1 Fig), excluding the possibility of off-target effects.

[Page 17, lines 15 - 17 (Materials and methods)]
In a selected experiment, U87 cells were transfected with a mouse M-Sec expression plasmid [10] using Lipofectamine 3000 (Invitrogen) [11].
[Page 20, lines 1 - 6 (Materials and methods)]
The antibodies used were as follows: anti-M-Sec (F-6; Santa Cruz Biotechnology), anti-p24 Gag (#65-004; BioAcademia), anti-gp120 Env (KD247; provided by S. Matsushita, Kumamoto University, Japan), anti-gp41 Env (2F5; obtained through BEI Resources, NIAID, NIH), anti-EXOC2 (#12751-1-AP; Proteintech), anti-EXOC3 (#14703-1-AP; Proteintech), anti-GFP (B-2; Santa Cruz Biotechnology), and anti-β-actin (EPR16769; Abcam).
[The legend of S1 Fig]
(A, B) The control or stable M-Sec knockdown (ΔM-Sec) U87 cells were transfected with the empty vector ("−") or the GFP-fused mouse M-Sec (GFP-mM-Sec) expression plasmid ("+"), and cultured for 3 days. In A, the cells were analyzed for their expression level of GFP-mM-Sec by western blotting. β-actin blot is the loading control. In B, the cells were infected with HIV-1, cultured for 2 days, and analyzed for Gag by immunofluorescence. The number of >0.25 μm Gag puncta in each Gag$^+$ cell is summarized (20 cells for each group). *n.s.*, not significant. $^*p < 0.05$.

Comment-4. U87 and 293 cells are not relevant HIV-1 target cells, and experiments with MDM are limited to the use of a poorly characterized M-Sec inhibitor. Depletion and rescue experiments should be performed with primary HIV-1 target cells to confirm the relevance of the observations.

Reply

According to the comment, we performed the transient knockdown experiment by employing the protocol with which we reported an efficient knockdown of apolipoprotein E in MDMs (PLoS Pathog 14 (11), e1007372, 2018) and the siRNA with which we reported an efficient knockdown of M-Sec in U87 cells (Retrovirology 17 (1), 20, 2020). As shown in S2 Fig, the knockdown of M-Sec was partial but detectable (S2C Fig), and the total number of Gag puncta in each Gag+ M-Sec knockdown cell was smaller than that in each Gag+ control cell (S2D Fig). Although more efficient knockdown is necessary for further analyses including viral infectivity assay, we added the following sentences to explain the result of Gag puncta formation and experimental procedures (underlined):
[Page 6, lines 16 - 18 (Results)]
The knockdown of M-Sec using siRNA was partial but detectable (S2C Fig), and the total number of Gag puncta in each Gag$^+$ M-Sec knockdown MDM was smaller than that in

each Gag+ control MDM (S2D Fig).

[Page 17, lines 24 - 27 (Materials and methods)]

In a selected experiment, the knockdown of M-Sec was performed using siRNA (Dharmacon) and RNAiMAX (Invitrogen), as described previously [47]. siRNAs used were non-targeting siRNA (pool #2; D-001206-14) and M-Sec-specific siRNA (#4; D-012267-17) [7].

[The legend of S2 Fig]

(C, D) In C, MDMs were transfected with the control siRNA or M-Sec-specific siRNA, cultured for 4 days, and analyzed for their expression level of M-Sec by western blotting. β-actin blot is the loading control. In D, MDMs were transfected with the control siRNA or M-Sec-specific siRNA, and cultured for 2 days. Then, the cells were infected with HIV-1, cultured for 2 days, and analyzed for Gag by immunofluorescence. The total number of Gag puncta (>0.03 μm) in each Gag+ cell is summarized (20 cells for each group). Data shown are a representative of three independent experiments.

Part III - Minor Issues

Comment. HIV-1 is primarily a T cell-tropic virus, but the authors state in the Discussion that M-Sec expression is low in primary CD4+ T cells and does not contribute to HIV-1 transmission. This is in agreement with the fact that certain human CD4+ T cell lines express no M-Sec at all, but are nevertheless highly permissive for HIV-1. In my opinion, it should be clarified in the title and abstract that the findings only apply to a subset of physiologically relevant target cells, if at all.

Reply

According to the comment, we added "macrophages" to the title and abstract as follows (underlined):

[Title]

M-Sec promotes the production of infectious HIV-1 virus through the exocyst complex in macrophages

[Abstract (the first sentence)]

We have demonstrated that the cellular protein M-Sec promotes the transmission of human immunodeficiency virus type 1 (HIV-1) in macrophages.
