# Supplementary material for: M-Sec promotes the production of infectious HIV-1 virus through the exocyst complex in macrophages
Source: PLoS Pathog. 2026 Jun 1;22(6):e1013717. doi: 10.1371/journal.ppat.1013717 (PMC13235924; doi:10.1371/journal.ppat.1013717)
Supplement: Attachment — Submitted filename: Reply (R2).pdf [file ppat.1013717.s028.pdf]

Reviewer #2

Part I - Summary

Mahmoud et al submit a revised version of their manuscript on the effect of M-Sec on the production and infectivity of HIV-1 particles. They made modifications in response to my previous comments and I will only address those comments that were not yet fully addressed.

Part II - Major Issues

Comment 1-1. I appreciate that the number of cells analyzed was increased from 15 and 20. The authors also state that they show one representative of three experiments conducted essentially throughout the manuscript. The rationale for this is not clear to me – showing the results from all three replicate experiments for all the panels listed would seem good practice and will significantly add to the robustness of the study.

Reply

According to the suggestion, we showed additional experimental sets in S1 Fig, S4 Fig, S6 Fig, S8 Fig, S9 Fig, S20 Fig, and S22 Fig.

Comment 1-2. Thanks for adding this helpful experiment. This shows that the effect of M-Sec ko on Gag clustering can be rescued but the questions was regarding particle production. Please add the particle production/infectivity data from this experiment.

Reply

According to the comment, we performed an additional experiment (the exogenous expression of mouse M-Sec in the human M-Sec knockdown U87 cells), and confirmed the rescue of infectivity (S13 Fig). To explain the result and experimental procedures, we added the following sentences (underlined):

[Page 9, lines 13 - 17 (Results)]

Importantly, when assessed using TZM-bl cells as target cells [15], viruses produced by the ΔM-Sec cells showed weaker infectivity than those by the control cells, even after the normalization to the RT activity (Fig 6E). <u>The weaker infectivity was rescued by the exogenous expression of mouse M-Sec in the ΔM-Sec cells (S13 Fig).</u>

[Page 17, lines 15 - 18 (Materials and methods)]

In a selected experiment, U87 cells were transfected with a mouse M-Sec expression plasmid [10] using Lipofectamine 3000 (Invitrogen) [11]. <u>The control or M-Sec knockdown U87 cells stably expressing mouse M-Sec were also established by culturing with 300 μg/mL G418.</u>

[The legend of S13 Fig]

The stable M-Sec knockdown U87 cells (ΔM-Sec), the ΔM-Sec U87 cells stably expressing mouse M-Sec (ΔM-Sec/mM-Sec), or the control U87 cells stably expressing mM-Sec (Control/mM-Sec) were infected with HIV-1, and cultured for 2 days. The supernatants were collected, and analyzed for viral infectivity using TZM-bl cells as the target cells (the viral input: 2 nU/mL reverse transcriptase activity). The infectivity is represented as a percentage relative to that of the ΔM-Sec U87 cells (n=3). *n.s.*, not significant. $^{*}p < 0.05$.

Comment 2-1. The authors are now much clearer regarding the limited magnitude of their effects but opt against conducting a replication experiments over multiple rounds. I strongly encourage the authors to add data from such an experiment. It is simple to do and will either shown that your effects potentiates or vanishes over multiple rounds, which is helpful information either way.

Reply

We appreciate the comment. In the previous study (reference #7), U87 cells co-expressing CD4 and CCR5 were transfected with either control or M-Sec-specific siRNA, cultured for 2 days, infected with the JRFL strain, and analyzed for the viral replication. As shown below (graph and images), the viral production gradually increased (~4 dpi) and became stable (5 ~ 6 dpi) due to the multiple infection-associated formation of severely-fused giant cells, and the inhibitory effect by the M-Sec knockdown was observed at 2 and 4 dpi, which is consistent with the present study. To explain the result and clarify that the knockdown of M-Sec in U87 cells retarded the HIV-1 production in the multiple-round short-term infection systems, we added the following phrase (underlined):

[Page 4, lines 14 - 21 (Introduction)]

Through an affinity-based chemical array screening, we previously discovered the small molecule compound NPD3064 [6], which bound M-Sec or M-Sec-containing molecular complex and inhibited the M-Sec-mediated TNT formation. We and others found that NPD3064 reduced not only the TNT formation but also HIV-1 production in monocyte-derived macrophages [6,9]. Consistent with this finding, the knockdown of M-Sec in the widely used HIV-1 target cell line U87 reduced the TNT formation and cell migration, and retarded the HIV-1 production in the multiple-round short-term infection systems [7].

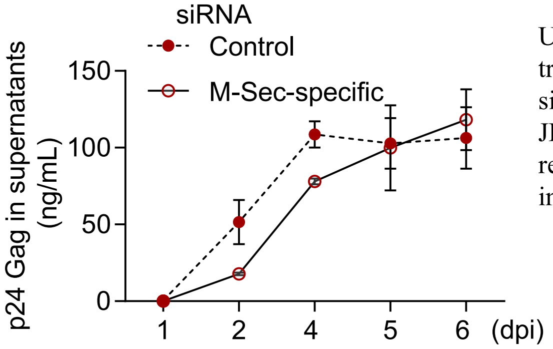

U87 cells co-expressing CD4 and CCR5 were transfected with either control or M-Sec-specific siRNA, cultured for 2 days, infected with the JRFL strain, and analyzed for the viral replication by p24 Gag ELISA. dpi, days post-infection.

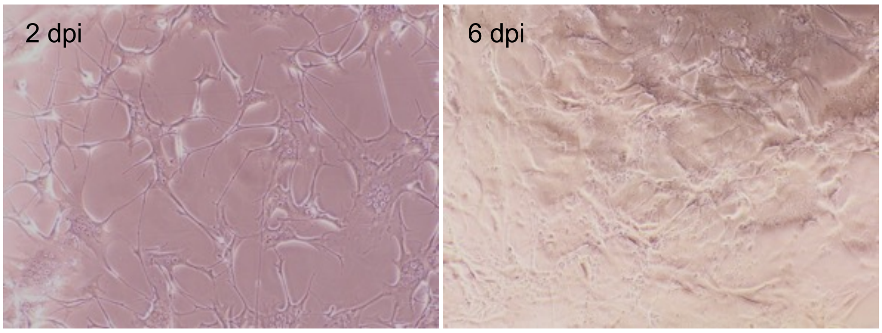

U87 cells co-expressing CD4 and CCR5 were infected with the JRFL strain, and cultured for 2 or 6 days.

Reviewer #3

Part I - Summary

In this revised paper, the authors have made a serious effort to address my concerns. For instance, they have performed a knockdown and rescue experiment in U87 cells to confirm that M-Sec induces the formation of Gag puncta in these cells. However, they did not examine whether the re-expression of M-Sec rescued Env incorporation and viral infectivity. I feel that these issues should also be examined in knockdown and rescue experiments, because the promotion of infectious particle production by M-Sec (not just the induction of Gag puncta) is what the paper really is about.

Reply

According to the comment, we performed an additional experiment (the exogenous expression of mouse M-Sec in the human M-Sec knockdown U87 cells), and confirmed the rescue of infectivity (S13 Fig). To explain the result and experimental procedures, we added the following sentences (underlined):

[Page 9, lines 13 - 17 (Results)]

Importantly, when assessed using TZM-bl cells as target cells [15], viruses produced by the ΔM-Sec cells showed weaker infectivity than those by the control cells, even after the normalization to the RT activity (Fig 6E). <u>The weaker infectivity was rescued by the exogenous expression of mouse M-Sec in the ΔM-Sec cells (S13 Fig).</u>

[Page 17, lines 15 - 18 (Materials and methods)]

In a selected experiment, U87 cells were transfected with a mouse M-Sec expression plasmid [10] using Lipofectamine 3000 (Invitrogen) [11]. <u>The control or M-Sec knockdown U87 cells stably expressing mouse M-Sec were also established by culturing with 300 μg/mL G418.</u>

[The legend of S13 Fig]

<u>The stable M-Sec knockdown U87 cells (ΔM-Sec), the ΔM-Sec U87 cells stably expressing mouse M-Sec (ΔM-Sec/mM-Sec), or the control U87 cells stably expressing mM-Sec (Control/mM-Sec) were infected with HIV-1, and cultured for 2 days. The supernatants were collected, and analyzed for viral infectivity using TZM-bl cells as the target cells (the viral input: 2 nU/mL reverse transcriptase activity). The infectivity is represented as a percentage relative to that of the ΔM-Sec U87 cells (n=3). *n.s.*, not significant. $^{*}p < 0.05$.</u>
